# 2Pipe starts with a question: matching you with the correct pipeline for MAG reconstruction

**DOI:** 10.1128/msystems.00844-25

**Published:** 2026-01-29

**Authors:** Jeferyd Yepes-García, Laurent Falquet

**Affiliations:** 1 Department of Biology, University of Fribourg 27211, Fribourg, Canton of Fribourg, Switzerland; 2 Swiss Institute of Bioinformatics 30489, Lausanne, Vaud, Switzerland; Medizinische Universitat Graz, Graz, Austria

**Keywords:** metagenomics, metagenome-assembled genome, pipeline benchmarking, workflow manager

## Abstract

Whole-genome sequencing has boosted our ability to explore microbial diversity by enabling the recovery of metagenome-assembled genomes (MAGs) directly from environmental DNA. As a result, the vast availability of sequencing data has prompted the development of numerous bioinformatics pipelines for MAG reconstruction, along with challenges to identify the most suitable pipeline to perform the analysis according to the user needs. This report briefly discusses the computational requirements of these pipelines; presents the variety of interfaces, workflow managers, and package managers they feature; and describes the typical modular structure. Also, it provides a compacted technical overview of 41 publicly available pipelines or platforms to build MAGs starting from short and/or long sequences. Moreover, recognizing the overwhelming number of factors to consider when selecting an appropriate pipeline, we introduce an interactive decision-support web application, 2Pipe, that helps users to identify a suitable workflow based on their input data characteristics, desired outcomes, and computational constraints. The tool presents a question-driven interface to customize the recommendation, a pipeline gallery to offer a summarized description, and a pipeline comparison based on key factors used for the questionnaire. Beyond this and foreseeing the release of novel pipelines in the near future, we include a quick form and detailed instructions for developers to append their workflow in the application. Altogether, this review and the application equip the researchers with a general outlook of the growing metagenomics pipeline landscape and guide the users toward deciding the workflow that best fits their expectations and infrastructure.

## INTRODUCTION

Metagenomics has advanced the study of microbial communities by diminishing the need for cultivation and enabling direct DNA sequencing from complex environments such as the human body, soil, or aquatic ecosystems (1). This has been possible thanks to the combination of high-quality and high-throughput sequencing technologies and recent advances in bioinformatics tools, increasing the scope and resolution at which the microbiota can be explored (2). Moreover, reconstructing metagenome-assembled genomes (MAGs) has enabled the genomic characterization of uncultured microorganisms, the discovery of previously unknown species, the inference of the community’s metabolic and functional potential, the establishing of ecological interactions, and the detection of evolutionary mechanisms (2, 3).

Considering the ecological importance of the MAGs, genomic criteria have been designed to determine whether a recovered bin (draft genome) truly represents a MAG or not. For instance, the Minimum Information about MAGs guidelines establish that MAGs can be classified into three quality tiers: high-quality drafts (HQ), medium-quality drafts (MQ), and low-quality drafts; the specific details regarding the genomic quality metrics used for this classification were introduced by Bowers et al. (4). MAGs can also be divided into species-assigned MAGs (SMAGs), that is, MAGs for which a species can be assigned, and hypothetical MAGs (HMAGs), that is, MAGs that are supposedly genomes of novel species, according to the genome heterogeneity spectrum proposed by Setubal (5).

**Fig 1:**
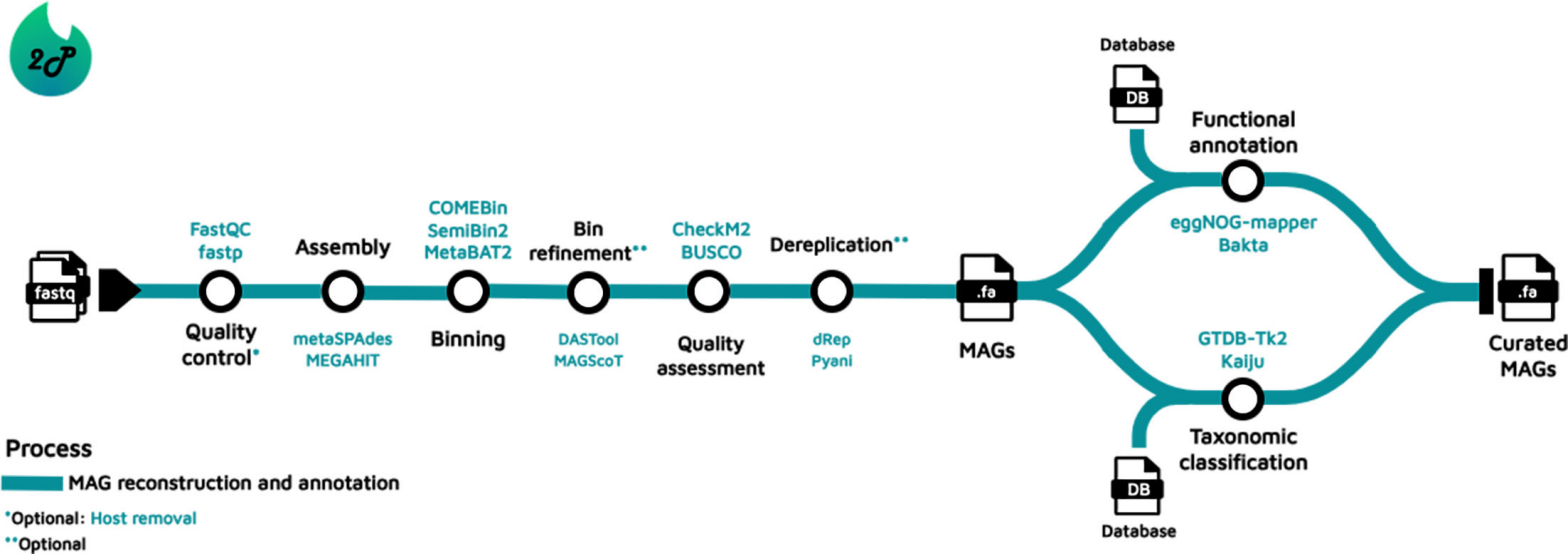
Usual bioinformatics workflow followed to perform MAG recovery, classification, and annotation. Some common tools incorporated by the pipelines are highlighted.

In a simplified manner, MAGs are obtained through bioinformatics pipelines that include quality control, assembling and binning the sequences, and the annotation of each recovered genome (6) (Fig. 1). These pipelines are then responsible for the correct MAG assembly and have a key role at extracting meaningful information about the structure and function of microbial communities (1). Through their orchestrated workflow, they simplify and standardize the common tasks that are required to achieve HQ MAGs, reducing the occurrence of manual errors by improving reproducibility (7). Nonetheless, pipeline choice may not be a trivial decision, given that it should be based on the alignment between user needs and workflow key factors such as the type of sequencing data they handle (short or long reads, or both), analytical functions (i.e., co-assembly, sequential co-assembly, taxonomic profiling, and eukaryotic recovery), and computational environment (e.g., availability of local resources, high-performance computing [HPC] infrastructure, or web-based tools). Therefore, pipeline selection can quickly become an overwhelming process and challenge researchers with a vast landscape of options, delaying the start of the analysis or even not obtaining the expected results since the incorrect workflow was chosen.

Here, we describe the general workflow followed by bioinformatics pipelines to recover MAGs directly from metagenomics data, discussing important aspects the pipelines feature, such as the tools they encompass and the type of data they can handle. We also succinctly highlight major considerations regarding pipeline execution, storage needs, and computational infrastructure. Likewise, we provide a compact overview of 41 publicly available pipelines, suites, or platforms that enable MAG reconstruction and/or annotation starting from short and/or long sequences. Finally, considering the main practical features of each pipeline and aiming at aiding researchers in navigating the ecosystem of workflows, we also introduce 2Pipe, a decision-support web application designed to match metagenomics community users with the most suitable MAG pipeline based on their input data, technical requirements, bioinformatics experience, and preferred interface.

## PIPELINE WORKFLOW, TOOLS, AND BENCHMARKS

The traditional computational workflow to build and annotate MAGs involves several steps (6); Fig. 1 introduces the general series of steps to potentially achieve MQ or HQ MAGs, along with some common software integrated by the pipelines. In brief, it begins with quality control, where low-quality reads and contaminants are removed (8, 9); when required, some pipelines include the option to discard host organism sequences (10). This is followed by the assembly step, where reads are extended to create contiguous sequences, also called contigs. The contigs are then grouped into bins that ideally represent individual genomes, based on the sequence composition and coverage patterns, among other genomic features (11). Optionally, the bins are subjected to a process of refinement when researchers consider it necessary (12, 13). Afterward, these bins are evaluated for common metrics such as completeness and contamination to assess their quality and hence determine whether they constitute MAGs or not, using the criteria previously mentioned (14). In some cases, the workflows can encompass dereplication tools or modules that attempt to curate the MAG set by clustering them according to their genomic similarity and thus selecting a representative MAG from each cluster (15). To conclude with the workflow, the MAGs are then taxonomically affiliated and functionally annotated to assign biological meaning, extracting insights related to their identity and potential roles within their microbial communities (16, 17). A detailed description of the tools for each step of the workflow is provided by Yang et al. (6), and Wajid et al. (18) present an overview of the typical analysis pipeline and software using an interesting music analogy.

We present on Table 1 the tools and third-party software for quality control, assembly, binning, refinement, taxonomic classification, and functional annotation that each of the pipeline documented here encompasses. Additionally, a detailed description of the main workflow for each of them can be found in File S1, where important technical considerations such as the type of input (short reads, long sequences, or both), tools employed at each step, advantages, limitations, and/or special features they depict are presented.

**TABLE 1:** Software and tools incorporated by each pipeline or web-based platform

No.	Pipeline/platform	Quality control preprocessing	Assembly^*a*^	Binning	Quality assessment	Bin refinement	Taxonomic annotation^*b*^	Functional annotation^*b*^	Other
1	Ancient DNA (19)	FastQC (20), fastp (8), and BBTools (21)	Bowtie2 and MEGAHIT (22)	CONCOCT (23), MaxBin (24), and MetaBAT (25)	CheckM (26)	DASTool (12)	GTDB-Tk (17)		mapDamage2 (27)
2	Anvi'o^*c*^ (28)	Illumina-utils (29)	metaSPAdes (30), MEGAHIT, and IBDA-UD (31)	MetaBAT2 (32), CONCOCT, MaxBin2 (33), and BinSanity (34)		DASTool	KrakenUniq (35) and Centrifuge (36)	DIAMOND (37) (NCBI Cluster of Orthologs Groups (COG) [38]), Pyrodigal (39), and HMMER (40)	
3	Aviary (41)	FastQC, Filtlong (42), NanoPack2 (43), and SingleM (44)	metaSPAdes, MEGAHIT, metaFlye (45), and Unicycler (46)	MetaBAT2, MetaBAT, MaxBin2, VAMB (47), CONCOCT, and Rosella (48)	CheckM, metaQUAST (49), and CoverM (50)	DASTool	GTDB-Tk	Prodigal (51) and DIAMOND (eggNOG [52])	Lorikeet (53)
4	BugBuster (54)	fastp and Bowtie2 (55)	MEGAHIT	METABAT2, SemiBin2 (56), and COMEBin (57)	CheckM2 (58)	MetaWRAP-native module (59)	GTDB-Tk2 (60)	Prodigal and MetaCerberus (61)	Kraken2 (62), Sourmash (63), and deepARG (64)
5	BV-BRC^*c*^ (65)	TrimGalore (66), BBTools, and BLAST (67)	metaSPAdes and MEGAHIT	PATRIC metagenome binning service (68)	EvalG and EvalCon (69)		RASTtk (70)		VIGOR4 (71) and Mat_Peptide (72)
6	DATMA (73)	Trimmomatic (9), FastQC, FLASH2 (74), and BWA (75)	metaSPAdes, Velvet (76), and MEGAHIT	CLAME (77)	CheckM		BLAST and Kaiju (78)	Prodigal and GeneMark (79)	Krona (80)
7	EasyMetagenome (81)	KneadData (82), HostPurge (81), and FastQC	metaSPAdes and MEGAHIT	MetaWRAP-native (59) module	CoverM and CheckM2	MetaWRAP-native module	GTDB-Tk2	MetaProdigal (83) and eggNOG-mapper (84)	dRep (85), Kraken2, Bracken (86), and HUMAnN3 (82)
8	EasyNanoMeta (87)	fastp, Minimap2 (88), SAMtools (89), Porechop (90), and BEDTools (91)	metaFlye, OPERA-MS (92), metaSPAdes, MetaPlatanus (93), and NextPolish (94)	SemiBin2, MetaBAT2, MaxBin2, CONCOCT, and VAMB	CheckM2		GTDB-Tk2 and PhyloPhlAn (95)	Prokka (96)	Kraken2 and Centrifuge
9	Eukfinder (97)	Bowtie2 and Trimmomatic	metaSPAdes	MyCC (98) and METAXA2 (99)			Centrifuge and PLAST (100)		
10	EURYALE (MEDUSA) (101, 102)	FastQC, fastp, Bowtie2, and MultiQC (103)	MEGAHIT				Kaiju and Kraken2	DIAMOND (NCBI nr [104])	Krona
11	Galaxy^*c*^ (105)	FastQC, Seqtk (106), and Trimmomatic	metaSPAdes	MaxBin2			GTDB-Tk2 and Contig Annotation Tool (CAT) (107)	Prokka	Kraken (108)
12	GEN-ERA (109)	fastp and FastQC	SPAdes (110), metaSPAdes, Canu (111), metaFlye, Pilon (112), and RagTag (113)	MetaBAT2 and CONCOCT	CheckM, GUNC (114), CheckM2, EukCC (115), BUSCO (116), Physeter (117), Kraken, and QUAST (118)		AMAW (119), BRAKER2 (120), and GTDB-Tk	Prodigal, Mantis (121), and Anvi'o scripts (Kyoto Encyclopedia of Genes and Genomes, KEGG [122])	OrthoFinder (123)
13	HiFi-MAG (124)			MetaBAT2 and SemiBin2	CheckM2	DASTool	GTDB-Tk2		
14	IDseq^*c*^ (125)	Trimmomatic, STAR (126), Bowtie2, and CD-HIT (127)	SPAdes and Bowtie2				GSNAPL (128) and RAPsearch2 (129)		
15	IMG/M^*c*^ (130)			SemiBin2	CheckM		GTDB-Tk	Prodigal, GeneMarkS-2 (131), and HMMER (NCBI COG, Pfam [132], and TIGRFAMs [133])	EukCC, SignalP (134), and TMHMM (135)
16	JAMS (136)	Trimmomatic and Bowtie2	MEGAHIT and SPAdes				Kraken2	Prokka and InterProScan (137)	Samtools and BEDTools
17	KBase^*c*^ (138)	FastQC, Trimmomatic, and Cutadapt (139)	metaSPAdes, MEGAHIT, and IBDA-UD	MetaBAT2, CONCOCT, and MaxBin2	CheckM	DASTool	RASTtk and GTDB-Tk	Prokka, dbCAN3 (140), and DRAM (141)	OMEGGA (142), ModelSEED2 (143), Kaiju, FastANI (144), dRep, FastTree2 (145), and Muscle5 (146)
18	MAGNETO (147)	fastp, Bowtie2, and FastQscreen (148)	MEGAHIT and Simka (149)	MetaBAT2	CheckM		GTDB-Tk,	Prodigal, Linclust (150), CD-HIT, eggNOG-mapper	mOTUs (151) and dRep
19	MAGO (152)	FastQC and fastp	metaSPAdes, MEGAHIT, and IBDA-UD	MaxBin2, MetaBAT, CONCOCT, and BinSanity	CheckM		GTDB-Tk	Prokka	Roary (153), ezTree (154), and FastANI
20	Mapler (155)	FastQC	metaMDBG (156), hifiasm (157), metaFlye, OPERA-MS, and Minimap2	MetaBAT2	CheckM2 and metaQUAST		GTDB-Tk2 and Kraken2		KAT (158)
21	MetaGEM (159)	fastp	MEGAHIT and BWA	MetaBAT2, CONCOCT, and MaxBin2	MetaWRAP–native module		GTDB-Tk	Prokka	Roary, CarveMe (160), SMETANA (161), MEMOTE (162), and GRiD (163)
22	MetaGenePipe (164)	Trimmomatic, TrimGalore, and FastQC	MEGAHIT				DIAMOND (SwissProt [165])	Prodigal and HMMER (166) (KOfam [167])	BLAST
23	Metagenome-Atlas (168)	BBTools	MEGAHIT and metaSPAdes	MetaBAT2, MaxBin2, and VAMB	BUSCO, CheckM, and CheckM2	DASTool	GTDB-Tk	Prodigal, eggNOG, and Distilled and Refined Annotation of Metabolism pipeline (DRAM)	dRep
24	Metagenomics- Toolkit (169)	fastp, Porechop, Filtlong, NanoPack2, KMC (170), and Nonpareil (171)	metaFlye, metaSPAdes, MEGAHIT, and Assembler Resource Estimator (169)	MetaBAT2, MetaCoAG (172), and MetaBinner (173)	CheckM	MAGScoT (13)	MMSeqs2 taxonomy (174) and GTDB-Tk2	Prodigal, Prokka, and RGI (175)	CarveMe, SMETANA, MEMOTE, gapseq (176), Pyani (177), and SANS (178)
25	Metaphor (179)	FastQC, fastp, and MultiQC	MEGAHIT	VAMB, MetaBAT2, and CONCOCT	metaQUAST	DASTool	DIAMOND (NCBI COG)	Prodigal and Prokka	
26	metagWGS (180)	FastQC, Cutadapt, Sickle (181), SAMtools, and BWA	metaSPAdes, MEGAHIT, hifiasm, and metaFlye	MetaBAT2, CONCOCT, and MaxBin2	metaQUAST	Binette (182)	GTDB-Tk2	Prodigal and eggNOG- mapper	dRep and Kaiju
27	MetaWRAP (59)	FastQC and TrimGalore	metaSPAdes and MEGAHIT	MetaBAT2, CONCOCT, and MaxBin2	CheckM	MetaWRAP-native module	Kraken and BLAST	Prokka	Kraken and Blobology (183)
28	MG-TK (184)	Trimmomatic, Porechop, Kraken, Kraken2, and SDM (185)	SPAdes, MEGAHIT, Flye (186), and metaMDBG	MetaBAT2, SemiBin2, and MetaDecoder (187)	CheckM and CheckM2		GTDB-Tk	Prodigal and DIAMOND (KEGG Carbohydrate-Active enZYmes, CAZy [188] and eggNOG)	mOTUs2 (189), MetaPhlAn (190), Freebayes (191), riboFinder (192), and BCFtools (89)
29	MGnify^*c*^ (193)	Trimmomatic and Biopython (194)	metaSPAdes				DIAMOND (UniRef90 [195])	Prodigal, FragGeneScan (196), InterProScan, eggNOG- mapper, and HMMER (40)	mOTUs2 and antiSMASH (197)
30	MOSHPIT^*c*^ (198)	Cutadapt and Bowtie2	SPAdes and MEGAHIT	MetaBAT2	QUAST and BUSCO	Sourmash	Kraken2 and Kaiju	eggNOG- mapper and DIAMOND (eggNOG and CAZy)	
31	MUFFIN (199)	fastp and Filtlong	SPAdes, Flye, and Unicycler	MetaBAT2, CONCOCT, and MaxBin2	CheckM	MetaWRAP-native module	Sourmash (Genome Taxonomy Database, GTDB [200])	eggNOG- mapper	Salmon (201) and Trinity (202)
32	NanoPhase (203)	Filtlong	metaFlye, Racon (204), and medaka (205)	MetaBAT2 and MaxBin2	CheckM and QUAST	MetaWRAP-native module	GTDB-Tk	Prodigal and DIAMOND (UniProtKB [206])	
33	nf-core/mag (207)	fastp, AdapterRemoval (208), Bowtie2, BBTools, Trimmomatic, FastQC, Porechop, Filtlong, and NanoPack2	MEGAHIT, metaSPAdes, Flye, metaMDBG, and hybridSPAdes (209)	MetaBAT2, CONCOCT, and MaxBin2	BUSCO, CheckM, CheckM2, GUNC, and QUAST	DASTool	GTDB-Tk2 and CAT	Prodigal, Prokka, and MetaEuk (210)	Kraken2, MultiQC, Centrifuge, PyDamage (211) geNomad (212), and Tiara (213)
34	ngs-preprocess MpGAp Bacannot (214)	Porechop, Nanopack2, pycoQC (215), and fastp	SPAdes, Flye, Canu, Unicycler, Shovill (216), HASLR (217), Raven (218), Shasta (219), wtdbg2 (220), and Pilon					Prokka, antiSMASH, KofamScan (167), KEGGDecoder (221), Bakta (16), and Barrnap (222)	AMRFinderPlus (223), CARD-RGI, BEDTools, Phigaro (224), VFDB (225), PlasmidFinder (226), MLST (227), Platon (228), PHASTER (229), ARGminer (230), and ResFinder (231)
35	nIMP3 (232)	BWA, Samtools, BBTools, FastQC, Kraken2, and SortMeRNA (233)	MEGAHIT						mOTUs, MultiQC, MetaPhlAn4 (82), Salmon, gffquant (234), and kallisto (235)
36	SnakeMAGs (236)	Illumina-utils, Trimmomatic, and Bowtie2	MEGAHIT	MetaBAT2	CheckM, GUNC, and CoverM		GTDB-Tk2		
37	SPIRE (237)	NGLess (238)	MEGAHIT, BWA, and Samtools	MetaBAT2	CheckM2 and GUNC		GTDB-Tk2	Prodigal and eggNOG-mapper	Barrnap, RGI (175), ABRicate (239) (MEGARes [240] and VFDB), Seqtk, Macrel (241), and Mash (242)
38	SqueezeMeta (243)	PRINSEQ 244, Trimmomatic, and SAMtools	MEGAHIT, SPAdes, Canu, and Flye	MetaBAT2, CONCOCT, and MaxBin2	CheckM, CheckM2, and CompareM 245	DASTool	GTDB-Tk2	Prodigal, MUMmer 246, HMMER, and Barrnap	DIAMOND (NCBI COG, KEGG), SQMtools 247 , and POGENOM 248
39	Sunbeam (249)	Trimmomatic, Cutadapt, Komplexity (249), and BWA	MEGAHIT					Prodigal, BLAST, and DIAMOND	Kraken
40	VEBA (250)	KneadData, fastp, BBTools, Bowtie2, NanoPack2, and Minimap2	metaSPAdes, SPAdes, rnaSPAdes (251), MEGAHIT, Flye, and metaFlye	MetaBAT2, CONCOCT, MaxBin2, and SemiBin2	CheckM2, Tiara, CheckV (252), BUSCO, and CoverM	Binette	GTDB-Tk2, MetaEuk, geNomad, and VirFinder (253)	Prodigal, DIAMOND (UniRef50/90, MIBiG [254], VFDB, and CAZy) HMMER (Pfam, NCBIfam-AMR [223], AntiFam [255], and KOfam), and MicrobeAnnotator (256)	antiSMASH, Muscle5, FastTree2, FastANI, sylph (257), and HUMAnN3
41	WGSA2+/LoRA^*c*^ (258)	KneadData, fastp, and Kraken2	metaSPAdes, metaFlye, MiniMap2, and Samtools	MetaBAT2	CheckM and CheckM2		GTDB-Tk2	Prodigal, eggNOG- mapper, and MinPath (259)	SortMeRNA, Krona, Trinity, and AMRFinderPlus

^*a*^ Not all the tools included here are assemblers; some of them are alignment or polishing tools that the pipeline’s assembly module includes.

^*b*^ If a database is necessary, it is mentioned in parentheses.

^*c*^ We describe here the main workflow to recover MAGs published on these platforms or suites. However, they may offer many more services, tools or pipelines to meet any other need that the users demand.

As previously mentioned, the MAG reconstruction workflow is triggered with the quality control of the raw reads to ensure the accuracy and integrity of downstream analyses. Usually, the reads received from the sequencing facility contain sequencing errors, low-quality bases, adapters, and contaminant sequences (e.g., host or environment DNA) that can lead to fragmented assemblies or chimeric bins if not properly removed (6, 10). These issues are addressed by filtering and trimming, if required, the raw reads using tools like Trimmomatic (9), fastp (8), Cutadapt (139), or BBTools (21). In the case of contamination removal, tools such as KneadData (82), Bowtie2 (55), Minimap2 (88), BWA (75), or Kraken (either v1 or v2) (62, 108) are commonly used to screen and remove host-derived or non-target reads. For long-read data (Oxford Nanopore known as ONT or Pacific Biosciences known as PacBio), Filtlong (42), Nanofilt (43), and Porechop (90) are used for length filtering, quality trimming, and adapter removal. The pipeline quality control and contamination removal modules are often complemented by FastQC (20) or MultiQC (103), the standard methods to evaluate the overall quality and report it; NanoPack2 and pycoQC (215) provide detailed quality summaries for long reads. In a recent report, Gao et al. (10) compared many available tools for removing host contamination, namely, KneadData, Bowtie2, KMCP (260), BWA, KrakenUniq (35), and Kraken2, highlighting the superior performance depicted by Bowtie2 in terms of resource usage, while Kraken2 demonstrated the shortest execution times; the accuracy of Bowtie2, KneadData, and BWA outperformed the rest of the tools.

Furthermore, the assembly step represents the core of the process since it reconstructs longer contiguous sequences from the high-quality reads. Notably, assembling metagenomics data sets faces complex challenges due to varying species abundance, uneven coverage, and the presence of closely related organisms (261). The short-read assemblers rely mainly on two strategies: overlap-layout-consensus, which aligns overlapping reads to build contigs, and the more widely used De Bruijn graph method, which decomposes reads into k-mers and represents them as nodes and edges in a graph (261). MEGAHIT (22), metaSPAdes (30), and IDBA-UD (31) are examples of tools that implement the De Bruijn graph approach, incorporating heuristics to address the coverage variation and strain complexity. In contrast, assemblers for long-read data such as metaFlye (45), Canu (111), and hifiasm (157) are designed to apply graph-based algorithms optimized for higher error rates and uneven depth. In some cases, hybrid strategies are employed, combining long reads for structural resolution with accurate short reads for initial graph assembly, as implemented in tools like OPERA-MS (92) and hybridSPAdes (209).

To this date, some authors have attempted to provide a comprehensive and unbiased benchmark of the most popular assemblers using different data sets that vary in complexity. For instance, Goussarov et al. (262) developed a comparison among short, long, and hybrid assemblers using a complex mock metagenome with more than 200 bacterial strains, demonstrating that metaSPAdes can achieve superior performance in terms of assembly fragmentation and chimerism when using Illumina reads, while Canu depicted the best metrics (chimerism and fragmentation) for ONT data. A similar conclusion regarding short-read assemblers was presented by Meyer et al. (263), where although MEGAHIT and metaSPAdes showed similar performance, metaSPAdes delivers fewer fragmented assemblies using simulated mouse gut sequences that enclosed more than 540 species. During the analysis of data sets enclosing mixed real metagenomic reads and reads from known genomes, Wang et al. (264) reported MEGAHIT as the most efficient assembler, while metaSPAdes outperformed MEGAHIT, IDBA-UD, and Faucet (265) in terms of integrity and continuity at the species level, and it showed the overall best performance at the strain level.

In the case of hybrid assembly, Brown et al. (266) showed boosted contiguity and reduced assembly errors with either hybridSPAdes or OPERA-MS, although yielding frequent misassemblies during *in silico* spike-in experiments using real and simulated reads. Nevertheless, assemblies obtained with these hybrid same tools were less complete and more fragmented than long-read only assemblies using the same data set of more than 200 bacterial strains mentioned above (262). As a result, Goussarov et al. suggest constructing the assembly using long reads complemented with short-read polishing, when the coverage is sufficient.

Accompanying the core of the pipelines, binning tools also represent an important step to reconstruct as accurately as possible the genomes present in the microbial communities. Classical binning strategies can be divided into different categories: (i) algorithms based on the genomic composition (mainly k-mer frequencies and GC content), (ii) approaches using read depth (coverage) profiles across multiple samples to link contigs with similar abundance patterns, and (iii) combined strategies that integrate both sequence composition and coverage signals (6). Classical tools based on these strategies such as MetaBAT2 (32), MaxBin2 (33), and CONCOCT (23) have been widely incorporated into the workflows given their efficiency and robustness. Nevertheless, more recent methods leverage machine learning and semi-supervised approaches to improve the resolution in more complex environments such as soil or ocean (267). SemiBin2 (56) represents an example of these recent strategies as it uses deep learning with semi-supervised contrastive learning to incorporate both intrinsic sequence information and external reference genomes. Another example is represented by COMEBin (57), which employs graph neural networks to integrate contrastive multiview representation learning, coverage, and a clustering algorithm.

Similar to the assembly case, there have been efforts to benchmark the performance of the available binning tools. In a recent report, Han et al. (11) used different combinations of short, long, and hybrid data to compare the outcomes from 10 binners, finding that deep-learning-based tools (COMEBin and SemiBin2) were almost always among the top three high-performance binners regardless of the combination of the contig provenance. Through comparisons among less tools, Cansdale & Chong (268) showed that CONCOCT generated more high-quality bins than MetaBAT2 using a simple gut metagenome, while Meyer et al. (263) reported homogeneous results among CONCOCT, MetaBAT2, and MaxBin2, with MAG completeness slightly increased by CONCOCT at the expense of genome purity. Contrastingly, Groopm2 (269) and MetaBAT2 provided the best performance metrics in recall, purity, and the number of high-quality genome bins at recovering MAGs from Critical Assessment of Metagenome Interpretation (CAMI) data sets (270). In addition, Yepes-García and Falquet (271) used environmental metagenomics samples (rice soil) to show how MetaBinner stands out for the greater number of bins recovered as compared with MetaBAT2 and SemiBin2, albeit only 10% of these were at least MQ MAGs.

Moreover, the inclusion (or enabling) of tools within the workflows to recover a non-redundant and high-quality MAG set is determinant. Several pipelines incorporate bin refinement modules or tools to improve the quality of the bin set as they reduce contamination, increase completeness, and may recover mis-binned contigs (12, 13, 85). The tools in charge of this task take as input the bins from different binning software to provide the best possible version of each bin and potential MAG. Among the existing tools for bin refinement, MAGScoT (13) is claimed by the developers as the piece of software with the best performance, as compared to DASTool (12) and the MetaWRAP-binning module (59), in terms of MAG quantity and quality using simulated marine and human gut data sets. Nonetheless, Han et al. (11) showed how MetaWRAP achieved the highest rank score (custom ranking score developed for the study) followed closely by MAGScoT, although this former tool demanded 10 times less memory and carried the bin refinement in one-tenth of a fraction of the time required by MetaWRAP.

Contamination estimation tools aid in the main goal of ensuring the reliability of the MAGs, with representative tools such as CheckM (26), BUSCO (116), and CheckM2 (58) that infer completeness and contamination based on single-copy marker genes from specific lineages or deep learning models. Notwithstanding, a benchmarking study (14) showed that CheckM may underestimate contamination, mainly if sequences from distantly related taxa are present, as it reported contamination values between 1% and 2% when the true contamination introduced by the researchers was 11%. In contrast, in the same study, the authors found that tools integrating phylogenomic signals or read classification strategies like GUNC (114), Kraken2 (62), Physeter (117), and Forty-Two (272) achieved contamination estimations closer to the true values and performed overall better at detecting inter-domain contamination. Further, within the CheckM2 paper itself, the developers demonstrated its greater accuracy to detect genome contamination conferred by unusual lineages and to predict genome completeness.

Similarly, some pipelines could include dereplication strategies after quality assessment, typically based on Average Nucleotide Identity with the aim of curating the MAG set and selecting the *best* representative MAG in each cluster of MAGs. Nonetheless, enabling the execution of these dereplication tools (85, 144, 177), as well as the parameter configuration, should always be thought thoroughly as discussed by Evans and Denef (15), who analyzed the advantages and drawbacks of running de-replication procedures. Briefly, these authors highlighted how dereplication maintains high quality of genomic databases and enhances coverage pattern estimations; however, dereplication may lead to a loss of information on variability in the auxiliary gene content among representatives from the same species.

One of the final stages when building MAGs is represented by reporting the taxonomic affiliation of each genome. The most common tool included within the workflows (Table 1) is GTDB-Tk (17) since it demonstrated that its phylogeny-based approach achieves high agreement (around 90%) with manually curated classifications in the GTDB, while GTDB-Tk v2 (GTDB-Tk2) is further optimized to reduce memory requirements without compromising the accuracy. Beyond this, the report describing the capabilities of CAT and BAT (107) included a benchmark against GTDB-Tk that demonstrated very similar performance as BAT and GTDB-Tk provided the same final MAG annotations.

Other classifiers not particularly designed to annotate MAGs can be included within the workflows such as MetaPhlAn4 (190), Kraken (108), Kraken2 (62), Centrifuge (36), and Kaiju (78) through the re-formatting of the draft genomes to make them suitable as input for these tools. There have been several efforts to benchmark taxonomic classifiers in a wide variety of scenarios and using different types of data (10, 273–279); however, these studies contrasting their performance and precision have shown variable results. For instance, Kraken2 in combination with Bracken exhibited superior precision, sensitivity, F1 score, and overall sequence classification of a custom *in silico* mock community within a comparison against MetaPhlAn and Kaiju (273); similar results were described by Timilsina et al. (274), who reported the highest accuracy and broad sensitivity achieved by Kraken2/Bracken (86) in simulated microbial communities as compared against MetaPhlAn4 and Centrifuge. Meanwhile, Irankhah et al. (275) observed how MetaPhlAn4 exhibited higher precision in identifying species in a simulated data set, outperforming Kraken2, Bracken, and Centrifuge. In contrast, when attempting to classify long reads (ONT), Kraken2 and Centrifuge demonstrated low to very low precision for all defined mock communities considered in the study (276). Similarly, Centrifuge depicted the worst performance at classifying sequences belonging to a mock community built from human fecal samples, within the study that introduced the tool DeepMicrobes (277).

To complete the final stages of the MAG reconstruction, functional annotation serves to reveal metabolic potential and ecological roles of microbial communities, with a remarkably high number of options available (280). The selection of these tools depends on the study goal, and it is usually a conscious decision made by the researchers. For more than 10 years, Prokka (96) has remained as standard for rapid genome annotation, predicting coding sequences, rRNAs, and tRNAs and assigning functions through curated databases. Nevertheless, more elaborated tools like eggNOG-mapper (84) have emerged to provide large-scale functional annotation, and the DRAM pipeline (141) offers detailed metabolic summaries. Web-based systems like RASTtk (70) (implemented within the Bacterial and Viral Bioinformatics Resource Center, BV-BRC [65]) and MGnify (193) can achieve quick and reliable annotations, while for specialized functional insights, tools like antiSMASH (197), KOfamKOALA (167), and dbCAN3 (140) are often incorporated into the workflows.

As shown on Table 1, taxonomic and functional annotation steps heavily rely on existing databases, highlighting the importance of these information resources. In the case of taxonomic classification, the GTDB (200) provides a phylogenetically consistent framework for prokaryotic and archaeal taxonomy, while nucleotide and protein repositories like UniRef (195) and Swiss-Prot (165) offer curated sequences that serve reliable standards for accurate assignments. On the functional prediction side, the KEGG (122) and its ortholog collection (KOfam [167]) enables the reconstruction of metabolic pathways, while Pfam (132) catalogs protein domains and families that help identify conserved protein functions. In the same sense, the database for evolutionary genealogy of genes: non-supervised Orthologous Groups (eggNOG) (52) covers orthologous groups linked to functional categories including COG (38), KEGG, and Gene Ontology terms (281). Other specialized databases are represented by the CAZy (188) and the database of proteolytic enzymes, their substrates, and inhibitors (MEROPS) (282). Please note that this is not a comprehensive review, and hence we suggest further reading of the works by Zeller and Huson (283) and Lin et al. (280), who explored and compared computational methods and classification systems, including databases, for protein function prediction.

Finally, benchmarking entire pipelines can be more challenging as they include many pieces of software which difficults setting a groundline for comparisons. Notwithstanding, there are a few works where the whole pipeline execution has been benchmarked, for instance, Churcheward et al. (147), who tested their pipeline performance (MAGNETO) against similar workflows such as nf-core/mag, Metagenome-Atlas, and MetaWRAP. These authors recovered a superior number of HQ MAGs from human gut microbiomes (Integrative Human Microbiome Project) through MetaWRAP operated in either single-assembly with single binning or co-assembly with a co-binning approach (see the next section for a detailed explanation of these approaches). Meanwhile, Yepes-García and Falquet (271), starting from sequences belonging to a mock community, depicted slight differences in terms of genome completeness, contamination, and number of MAGs taxonomically annotated at species level among MetaWRAP, nf-core/mag, SnakeMAGs, and Metagenome-Atlas. nf-core/mag reached the highest percentages of MQ and HQ MAGs, whilst DATMA, also included in this study, performed poorly as only 40% of the MAGs were assigned a proper taxonomic classification and not a single MQ or HQ MAG was recovered.

## PRACTICAL AND TECHNICAL CONSIDERATIONS FOR PIPELINE EXECUTION

As high-throughput sequencing technologies have grown in the past years, the availability of MAG-centered pipelines has been quickly expanded to handle and integrate different data types and computational strategies (169, 180, 250). Specifically, recent pipelines have been designed or have evolved to assemble and bin short reads (normally Illumina), long reads (mainly ONT and PacBio), or a blend of both technologies to maximize base calling, depth, contiguity, and structural information (180, 250). Short reads synthesized through DNA nanoball sequencing (284) or long reads derived from CycloneSEQ (285) can be eventually processed by some pipelines (207, 214). Differences or similarities among these MAG-reconstruction approaches based on the type of sequence used as input have been studied by Goussarov et al. (262), and Kim et al. (286) analyzed the variations in terms of genome recovery between Illumina and MGI platforms.

Among the several tools that compose a pipeline (Fig. 1), assembly and binning tools are mainly responsible for the scaling up in the hardware demands, especially when handling data sets with several samples encompassing millions of short-read sequences (6). Moreover, these tools can be executed in different configurations such as co-assembly and co-binning, as these strategies can increase the overall MAG recovery rate and quality (287). Briefly, co-assembly refers to the possibility of performing the metagenome assembly after merging user-specified samples to enhance the coverage, capturing a higher fraction of the diversity (287), while co-binning establishes the possibility of binning contigs using coverage information across multiple samples simultaneously after single or co-assembly (11). Co-binning is advantageous at exploring coverage across samples and improving separation of closely related genomes (47). Despite the desirable benefits co-assembly can bring to the analysis, it is computationally intensive and increases the probability of generating fragmented assemblies (147), although sequential co-assembly has emerged recently as an efficient alternative that enhances both time and memory requirements by the assembler (288). Similarly, co-binning can be sensitive to uneven sequencing depth, requires high-quality coverage profiles, and can be affected by low diversity among samples (147). Vosloo et al. (287) and Han et al. (11) have demonstrated how superior performance can be achieved by applying co-assembly and/or co-binning.

On the other hand, the workflow execution varies in terms of computational demands, where small-scale data sets can be processed on high-end workstations, while large or complex metagenomes often require access to HPC clusters or cloud-based environments (Azure, Amazon Web Services or AWS, Google Cloud, and Terra, among others). Beyond sample-specific computational requirements, and as mentioned before, most metagenomics pipelines rely on external reference databases to perform taxonomic classification, functional annotation, and quality assessment of MAGs. Commonly used databases, namely, RefSeq (289), GTDB, UniProt (206), KEGG, and eggNOG, are large and require substantial local storage that ranges from tens to hundreds of gigabytes. For instance, the latest GTDB release (R226) exceeds 140 GB, while comprehensive functional annotation pipelines like DRAM can demand up to 500 GB to exploit its full potential. Being so, MAG building is a demanding process that needs adequate disk space, CPU capacity, and memory availability.

For researchers without access to HPC resources, web-based platforms such as KBase (290), MGnify (193), Galaxy (105), and BV-BRC (65), among others, can assist them by carrying out analysis execution in their servers. In addition, these platforms aid users without a strong experience in command line interface (CLI) interaction since they provide user-friendly interfaces where users can upload raw reads and run predefined workflows. As a result, these platforms eliminate the need for CLI proficiency and offer built-in visualization applications and databases for downstream interpretation; a complete landscape of web-based applications is compiled by Achudhan et al. (291) and Chivian et al. (138).

Furthermore, given the MAG pipeline evolution in complexity, involving multiple tools, dependencies, and steps, the use of workflow managers has become the standard to ensure reproducibility, scalability, and portability (292). Specifically, workflow managers ease pipeline step definition in a modular and automated architecture to orchestrate entire analyses, tracking software versions, managing intermediate files, restarting the process if interrupted, handling multiple samples as input, and enabling parallel processing in a reproducible manner. Some representatives of these helpful orchestrators are Snakemake (293), Nextflow (294), and Workflow Definition Language (WDL) (295) whose design, implementation, benefits, and scope have been reviewed in some reports (292–294, 296); also, important guidelines for pipeline design based on workflow managers have been published by Roach et al. (297), Reiter et al. (298), and Ahmed et al. (7). Advantageously, containerization platforms such as Docker, Singularity, and Seqera Containers, or package managers like Conda or the Python Package Index complement workflow orchestrators by offering a flexible and reproducible solution for software and dependency management (299). As a result, this combination allows users to run the analysis without system conflicts, specific versions of the software, and libraries.

In contrast, beyond the MAG assembly and annotation, some pipelines feature interesting options that complement the analysis and provide a wider understanding of the microbial community. The range of these special options is wide, and therefore they must be carefully selected. In this sense, read-based taxonomic profiling (1) is one of the most common offerings by the pipelines as this process does not rely on the main workflow and can be executed in parallel. Furthermore, some pipelines can incorporate tools or modules to recover viral or eukaryotic MAGs (250), and it is even possible to find pipelines mostly focused on this type of MAGs (97). Another popular extra option is represented by the possibility of establishing genome-scale metabolic models among the built MAGs (159, 169). However, in many cases, some workflows can be considered unique since they include options that no other pipeline encompasses. Examples of these rare features are the possibility to assemble plasmids (169), genotype recovery (41) , controlled resource allocation (169), and an alternative assembly and binning order, where the reads are first grouped (binning) and then assembled in batches (73).

On Table 2, we present a summarized overview of the technical features and methodological factors each workflow presents, and hence these same pipeline aspects are also the basis for the questionnaire presented on 2Pipe. Methodological factors include the ability to assemble short reads, long sequences, or both in a hybrid approach; the possibility to request a co-assembly and/or co-binning natively; whether the user can input multiple samples or not; if the pipeline includes a bin refinement tool; and special functionalities they may incorporate. In the same sense, technical features are described through factors like which kind of resources the user is planning to use for the pipeline execution, the interface they feel more comfortable working with, the workflow manager they expect to orchestrate the data flow, and the software/package technology management available within each workflow. We assigned one of the following (non-mutually exclusive) labels in order to classify them: short-read-centered or long-read-focused (if their main input is short or long reads), dual (if they can handle both long and short reads, but they do not perform hybrid assembly), hybrid (pipelines able to assemble short and long reads together), web-based (pipelines offered by online platforms or suites), or special (pipelines designed for a specific purpose).

**TABLE 2:** Technical and operational features for each pipeline or web-based platform

No.	Pipeline/ Platform	Category	Short reads	Long reads^*a*^	Hybrid assembly	Multiple samples	Co-assembly and/or co-binning^*b*^	Bin refinement	Infrastructure^*c*^	Interface^*d*^	Workflow manager	Software execution	Special features	Last update^*e*^	Number of citations^*e*^	License^*f*^
1	Ancient DNA (19)	Special	Yes	No	No	No	No	Yes	Local and HPC	CLI		Local	Ancient DNA identification	2024	0	Not specified
2	Anvi'o (28)	Short-read-centered	Yes	No	No	Yes	Yes	Yes	Local and HPC	CLI/graphical user interface (GUI)		Conda	Visualization module	2025	678	GNU GPL v3
3	Aviary (41)	Hybrid	Yes	Yes	Yes	Yes	No	Yes	Local, HPC, and CC	CLI	Snakemake	Conda	Genotype recovery	2025	Not found	GNU GPL v3
4	BugBuster (54)	Short-read-centered	Yes	No	No	Yes	No	Yes	Local, HPC, and CC	CLI	Nextflow	Docker	Taxonomic profiling and antimicrobial resistance gene prediction	2025	0	Not specified
5	BV-BRC (65)	Web-based	Yes	No	No	Yes	No	No	External	GUI		External	Taxonomic profiling and viral MAGs	2024	783	MIT License
6	DATMA (73)	Short-read-centered	Yes	No	No	No	No	No	Local and HPC	CLI	COMP Superscalar (300)	Local	Reads first grouped (binning) and assembled in batches	2020	4	GNU GPL v3
7	EasyMetagenome (81)	Short-read-centered	Yes	No	No	Yes	Yes	Yes	Local and HPC	CLI		Conda	Taxonomic profiling	2024	14	GNU GPL v3
8	EasyNanoMeta (87)	Long-read-focused	No	Yes (ONT)	Yes	Yes	No	No	Local and HPC	CLI		Conda, Singularity	Taxonomic profiling	2024	0	GNU GPL v3
9	Eukfinder (97)	Special	Yes	Yes	No	No	No	No	Local and HPC	CLI		Conda	Eukaryotic MAGs	2025	1	MIT License
10	EURYALE (MEDUSA) (101, 102)	Short-read-centered	Yes	No	No	Yes	No	No	Local, HPC, and CC	CLI	Nextflow	Conda, Singularity, Docker		2024	7	MIT License
11	Galaxy (105)	Web-based	Yes	Yes	Yes	No	No	Yes	External	GUI		External	Taxonomic profiling	2024	1168	Academic Free License v3
12	GEN-ERA (109)	Dual	Yes	Yes (ONT)	No	Yes	No	No	Local, HPC, and CC	CLI	Nextflow	Singularity	Metabolic modeling	2024	7	GNU GPL v3
13	HiFi-MAG (124)	Long-read-focused	No	Yes (PacBio)	No	Yes	No	Yes	Local, HPC, and CC	CLI	Snakemake	Conda		2025	8	BSD-3-Clause-Clear License
14	IDseq (125)	Web-based	Yes	Yes (ONT)	No	No	No	No	External	GUI		External	Viral MAGs	2025	347	MIT License
15	IM**G/**M (130)	Web-based	NA	NA	NA	No	No	No	External	GUI		External	Eukaryotic MAGs	2025	268	IMG Expert Review Submission Agreement
16	JAMS (136)	Short-read-centered	Yes	No	No	No	No	No	Local and HPC	CLI		Conda	Direct sample comparison	2025	7	GNU GPL v3
17	KBase (138)	Web-based	Yes	Yes	Yes	Yes	Yes	Yes	External	GUI		External	Taxonomic profiling and metabolic modeling	2024	63	MIT License
18	MAGNETO (147)	Short-read-centered	Yes	No	No	Yes	Yes	No	Local, HPC, and CC	CLI	Snakemake	Conda	Taxonomic profiling	2025	13	GNU GPL v3
19	MAGO (152)	Short-read-centered	Yes	No	No	No	No	Yes	Local and HPC	CLI		Singularity, Docker	Phylogenetic tree generation and pangenome analysis	2020	21	Creative Commons BY 4.0
20	Mapler (155)	Long-read-focused	No	Yes (PacBio)	No	Yes	No	No	Local, HPC, and CC	CLI	Snakemake	Conda	Visualization module	2025	0	GNU AGPL v3
21	MetaGEM (159)	Short-read-centered	Yes	No	No	Yes	No	Yes	Local, HPC, and CC	CLI	Snakemake	Conda	Eukaryotic MAGs and metabolic modeling	2023	99	MIT License
22	MetaGenePipe (164)	Short-read-centered	Yes	No	No	Yes	Yes	No	Local, HPC, and CC	CLI	WDL (295)	Singularity		2023	1	Apache License 2.0
23	Metagenome-Atlas (168)	Short-read-centered	Yes	No	Yes	Yes	Yes	Yes	Local, HPC, and CC	CLI	Snakemake	Conda		2024	159	BSD-3-Clause-Clear
24	Metagenomics- Toolkit (169)	Dual	Yes	Yes (ONT)	No	Yes	No	Yes	Local, HPC, and CC	CLI	Nextflow	Docker	Plasmid assembly, metabolic modeling and controlled resource allocation	2025	0	GNU AGPL v3
25	Metaphor (179)	Short-read-centered	Yes	No	No	Yes	Yes	Yes	Local, HPC, and CC	CLI	Snakemake	Conda	Visualization module	2024	13	MIT License
26	metagWGS (180)	Dual	Yes	Yes (PacBio)	No	Yes	Yes	Yes	Local, HPC, and CC	CLI	Nextflow	Singularity	Taxonomic profiling	2025	2	GNU GPL v3
27	MetaWRAP (59)	Short-read-centered	Yes	No	No	Yes	Yes	Yes	Local and HPC	CLI		Conda and Docker	Taxonomic profiling	2020	1917	MIT License
28	MG-TK (184)	Dual	Yes	No	No	Yes	Yes	No	Local and HPC	CLI		Conda	Taxonomic profiling and strain delineation	2025	99	GNU GPL v2
29	MGnify (193)	Web-based	Yes	Yes	Yes	Yes	Yes	No	External	GUI		External	Taxonomic profiling	2025	286	Apache License 2.0
30	MOSHPIT (198)	Short-read-centered	Yes	No	No	Yes	No	Yes	Local and HPC	CLI		Conda	Taxonomic profiling	2025	1	BSD-3-Clause-Clear
31	MUFFIN (199)	Hybrid pipelines	No	Yes (ONT)	Yes	Yes	No	Yes	Local, HPC, and CC	CLI	Nextflow	Conda, Docker, and Singularity	Metatranscriptome support	2022	34	GNU GPL v3
32	NanoPhase (203)	Long-read-focused	No	Yes (ONT)	Yes	No	No	Yes	Local and HPC	CLI		Conda		2023	73	MIT License
33	nf-core/mag (207)	Hybrid	Yes	Yes (ONT or PacBio)	Yes	Yes	Yes	Yes	Local, HPC, and CC	CLI	Nextflow	Conda, Docker, Singularity and Others	Ancient DNA identification	2025	57	MIT License
34	ngs-preprocess MpGAp Bacannot (214)	Hybrid	Yes	Yes	Yes	Yes	No	No	Local, HPC, and CC	CLI	Nextflow	Conda, Docker, Singularity	Antimicrobial resistance gene prediction, virulence factor annotation, and plasmid assembly	2025	2	GNU GPL v3
35	nIMP3 (232)	Short-read-centered	Yes	No	No	Yes	No	No	Local, HPC, and CC	CLI	Nextflow	Docker, Singularity	Metatranscriptome support and taxonomic profiling	2024	150	MIT License
36	SnakeMAGs (236)	Short-read-centered	Yes	No	No	Yes	No	No	Local, HPC, and CC	CLI	Snakemake	Conda		2024	6	CeCILL Free Software License Agreement v2.1
37	SPIRE (237)	Short-read centered	Yes	No	No	Yes	No	No	Local, HPC, and CC	CLI	Nextflow		Antimicrobial resistance gene prediction and virulence factor annotation	2025	41	MIT License
38	SqueezeMeta (243)	Hybrid	Yes	Yes	Yes	Yes	Yes	Yes	Local and HPC	CLI		Conda	Taxonomic profiling, metatranscriptome support, and visualization module	2025	400	GNU GPL v3
39	Sunbeam (249)	Short-read-centered	Yes	No	No	Yes	No	No	Local and HPC	CLI	Snakemake	Conda and Docker	Taxonomic profiling	2025	184	GNU GPL v3
40	VEBA (250)	Dual	Yes	Yes (ONT or PacBio)	No	Yes	Yes and pseudo- coassembly	Yes	Local and HPC	CLI	GenoPype (301)	Conda and Docker	Eukaryotic or viral MAGs, antimicrobial resistance gene prediction, and virulence factor annotation	2025	23	GNU AGPL v3
41	WGSA2+/LoRA (258)	Web-based	Yes	Yes (ONT or PacBio)	No	Yes	No	No	External and CC	GUI	AWS environment	External	Visualization module, metatranscriptome support, and antimicrobial resistance gene prediction	2025	138	CC0 1.0 Universal

^*a*^ Long reads: ONT, Oxford Nanopore Technology; PacBio, Pacific Biosciences.

^*b*^ Co-assembly and/or co-binning: it highlights if the pipeline counts with options to control co-assembly and/or co-binning.

^*c*^ Infrastructure: it refers to the computational infrastructure where the pipeline can be executed natively. HPC, high-performance cluster; CC, cloud computing; External, pipelines controlled by the platform or suite and use third-party resources.

^*d*^ Interface: CLI, command line interface; GUI, graphical user interface.

^*e*^ Last update and number of citations: at the moment of writing this report.

^*f*^ License: these licenses cover the pipeline code and platforms; the third-party software and tools they encompass may be covered by a different license.

## 2PIPE: IT STARTS WITH A QUESTION

Considering the pipeline landscape identified in this review, we have developed a decision-support application that concatenates most of the features described for each workflow. 2Pipe is an interactive web application designed to help researchers identify the most suitable metagenomics pipeline for reconstructing and annotating MAGs. 2Pipe can be used by users with different expertise levels and computational access, simplifying the often-complex selection process by mapping user needs to a curated database of available pipelines.

At the core of 2Pipe, there is a dynamic and question-driven interface that guides users step by step through a personalized questionnaire. This adaptive form collects information related to the methodological factors and technical features detailed on Table 2. Therefore, every response is used to assign a score to each pipeline based on the presence or absence of specific features that align with the user’s input. The recommendation system will then suggest the pipeline with the highest score, as well as the second “best hit” for the user to check in case the first option does not fulfill their requirements; these suggestions can be as well the starting point for the user to dig into the other sections of 2Pipe. It is worth mentioning that the scoring is weighted, and some features have prevalence as they are definitive for the pipeline suggestion. Specifically, all matching features presented in the questions add one point to the final score, excepting type of reads to analyze (2 points), the need for a GUI (3 points), and the requirement for external computational resources (3 points). These features are prioritized then, and the recommendation must reflect them as they cannot simply be bypassed with any other pipeline. The system also includes a protection for cases when the users do not provide at least three answers, asking them to restart the questionnaire. Likewise, in case of a tie among more than two pipelines, the recommendation system will show all of them with the respective matching features.

Aside from the accession to the questionnaire and the response-based recommendation at the end of this, 2Pipe as well encompasses a pipeline gallery, where a visual catalog is displayed, offering individual summaries of each pipeline and a direct access to the source code or to the publication that documents the pipeline. Additionally, 2Pipe makes available an interactive view of Table 2 that includes the possibility of filtering by each feature or by a combination of them, allowing users to directly tailor the search for the pipeline that best suits their needs; the displayed categories are the same key attributes the question-based suggestion system relies on. 2Pipe also incorporates the features presented in Table 1, assisting the user when comparing the pipelines beyond technical aspects. Also, these tools and external software are organized in a gallery that allows the user to match pipelines that use them, which is useful if the user is looking for a specific software combination that a specific pipeline can offer.

On the other hand, given the importance pipeline and tool benchmarking represents, 2Pipe provides an exclusive page where the reports cited in this work comparing performance and/or technical features are introduced. This page is divided into sections according to the tools benchmarked in the papers, namely, assemblers, binners, bin-refinement tools, contamination-estimation software, complete pipelines, workflow managers, and taxonomic classifiers. Moreover, we include sections for reviews, tutorials, and protocols for manual MAG reconstruction and key papers that set interesting discussions around MAG recovery.

The source code for 2Pipe is available at the repository https://github.com/jeffe107/2pipe, and foreseeing the possibility of new pipelines being released in the near future, we provide a quick form for developers to include their workflow into 2Pipe’s recommendation system, pipeline gallery, and table comparison. Also, at the GitHub repository, developers can find a simple template and detailed instructions for the inclusion of their pipeline through a pull request.

## CONCLUSION

The rapid evolution of sequencing technologies has broadened the availability of metagenomics data sets that demand bioinformatics tools adjusted to the user requirements to achieve cutting-edge analysis, including MAG reconstruction. As a result, in the past 10 years, a rise in the number of MAG reconstruction pipelines available has been observed, and the selection of the proper pipeline for the analysis has become an essential step during the execution of metagenomics projects. This review offers a compact description of 41 publicly available pipelines or platforms, with special focus on their capabilities and distinctive features to serve as a valuable resource for researchers navigating this overwhelming landscape. Beyond the scope of a classical review, we streamlined the selection process by introducing 2Pipe, an interactive decision-support web application that aligns the user needs with the most convenient workflow for their analysis and allows a general overview of the pipeline universe with its gallery and pipeline-comparison sections. Finally, this review and its accompanying application provide a unified framework that simplifies the decision-making process, releasing part of the burden and uncertainty when setting a metagenomics data analysis project.

## Data Availability

2Pipe is hosted under the domain https://2pipe.app/. The source code is available at https://github.com/jeffe107/2pipe, along with a template to include new pipelines. The quick form to add a new pipeline can be found at https://form.jotform.com/jeffe10789/2pipe-form. For version tracking, 2Pipe v.2.0 release has been deposited at Zenodo, and it can be followed with the identifier https://doi.org/10.5281/zenodo.17334924.

## SUPPLEMENTAL MATERIAL

File S1 Detailed summary description for each pipeline considered in this review.

ASM does not own the copyrights to Supplemental Material that may be linked to, or accessed through, an article. The authors have granted ASM a non-exclusive, world-wide license to publish the Supplemental Material files. Please contact the corresponding author directly for reuse.
