# Supplementary material for: 2Pipe starts with a question: matching you with the correct pipeline for MAG reconstruction
Source: mSystems. 2026 Jan 29;11(2):e00844-25. doi: 10.1128/msystems.00844-25 (PMC12915991; doi:10.1128/msystems.00844-25)
Supplement: File S1 — Detailed summary description for each pipeline considered in this review. [file msystems.00844-25-s0001.pdf]

2Pipe: It starts with a question. Matching you with the correct pipeline for MAG reconstruction

Jeferyd Yepes-García[a,b], Laurent Falquet[a,b,*]

[a]Department of Biology, University of Fribourg, Fribourg, Canton of Fribourg, 1700, Switzerland
[b]Swiss Institute of Bioinformatics, Lausanne, Vaud, 1015, Switzerland
[*]Correspondence should be addressed to L.F (laurent.falquet@unifr.ch)

Descriptive pipeline overview

Below we present a descriptive overview of the main workflow for each pipeline or platform, where important technical considerations such as the type of input (short reads, long reads or both), key tools employed at each step, advantages, limitations and/or special features they depict are documented.

1. Short-read centered pipelines

1.1 Anvi'o[1]

Anvi'o is a comprehensive modular platform for the analysis and visualization of microbial omics including, but not restricted to, metagenomics, metatranscriptomics and metapangenomics. Anvi'o is developed to be highly customizable through exchangeable programs (tools) that perform specific tasks, empowering the user with a wide range of tools to explore. Being so, a metagenomics workflow is proposed by the developers of the platforms that begins with short-read quality cleaning, proceeds to read assembly to be used for read recruitment (mapping), and finalizes contig annotation (functions, Hidden Markov Models, and taxonomy). Optionally, the user can achieve read taxonomic profiling with KrakenUniq[2], and more recently binning tools have been made available such as MetaBAT2[3], CONCOCT[4], MaxBin2[5] and BinSanity[6], as well as DASTool[7] as a refinement alternative. Nonetheless, the user must run the analysis manually, requiring them to account with some experience regarding software installation, execution and debugging. Moreover, although Anvi'o is in principle a command line tool, it incorporates a user-friendly graphical interface for data inspection and visualization that is commonly used for contig visualization.

1.2 BugBuster[8]

BugBuster is an automatic, modular, and reproducible Nextflow[9] (DSL2) workflow with specialized modules for taxonomic profiling and resistome characterization. Its workflow encompasses the following steps: initial reads processing for quality filtering and host contamination removal (Bowtie2[10]); taxonomic profiling at the read level using tools like Kraken2[11]/Bracken[12] or Sourmash[13]; and antibiotic resistance gene (ARG) prediction from reads using KARGA[14] and KARGVA[15]. The assembly is carried out with MEGAHIT[16], followed by taxonomic and functional annotation of contigs using BLAST[17], BlobTools[18], DeepARG[19], and MetaCerberus[20]. Afterwards, the contigs are binned with tools such as MetaBAT2[3], SemiBin2[21] and COMEBin[22], and refined them with a MetaWRAP[23]-native module; the quality is assessed with CheckM2[24], and the MAGs are taxonomically affiliated with GTDB-Tk2[25]. BugBuster is fully containerized (Docker) aiming at ensuring ease of installation, high reproducibility, and deployment across various computational environments. Moreover, BugBuster stands out given its inclusion of specific tools to characterize and quantify genes associated with antibiotic resistance.

1.3 DATMA[26]

DATMA (Distributed AuTomatic Metagenomic Assembly and annotation framework) is a pipeline focused on speed and automation, leveraging distributed computing for efficiency. As a

starting point, DATMA applies a quality filter with RAPPIFILT (customized tool developed for this pipeline), Trimmomatic[27] and FastQC[28], and if the input sequences are paired-end, it merges them using FLASH2[29] and ForceMerge. Following this procedure, this pipeline identifies and removes 16S rDNA sequences based on RFAM[30] (RNA sequence families), NCBI[31], Ribosomal Database Project (RDP)[32] and SILVA[33] to cluster the remaining sequences with CLAME[34]. The clusters (or bins in definition of the traditional workflow) generated then are assembled in batches by metaSPAdes[35], Velvet[36], and MEGAHIT[16] for a subsequent taxonomic annotation relying on BLAST[37] and Kaiju[38], as well as ORF prediction with Prodigal[39] and GeneMark[40]. To conclude with the analysis a detailed HTML report is generated with interactive Krona[41] plots for taxonomic visualization; this report integrates the 16S rDNA annotation (RDP Classified) along with the annotated bins. As inferred from the described workflow, DATMA performs an inverted approach to generate bins by first grouping the reads using CLAME and attempting to assemble only these groups individually afterwards. Further, this pipeline is wrapped by COMP Superscalar which facilitates the development and execution of parallel applications for distributed infrastructures such as clusters, cloud services and containerized platforms.

1.4 EasyMetagenome[42]

EasyMetagenome integrates a classical workflow starting with short reads to provide a de-replicated (dRep[43]) set of bins and pangenome analysis that relies on an Anvi'o module. The assembly is performed with MEGAHIT[16], a MetaWRAP[23] module is in charge of the binning task, CheckM2[24] controls the quality of the bins, and GTDB-Tk2[25] finalizes the execution by taxonomically annotating them. Notably, this pipeline performs functional annotation (GhostKOALA[44], eggNOG[45], dbCAN3[46]) and taxonomy assignment on the contigs after a pre-filtering step that generates a non-redundant gene set. EasyMetagenome uses Conda environments to assure reproducibility, the user can input multi-sample data, although it is not orchestrated by any workflow manager. As special remarks, it carries out a taxonomic profiling (MetaPhlAn[47], HUMAnN3[48], Kraken2[11]) of the post-filtered (KneadData) reads, and the functional annotation of the gene set is expanded to identify virulence factors (VFDB[49]) and antibiotic resistant genes (CARD[50]).

1.5 EURYALE (MEDUSA)[51,52]

EURYALE is a Nextflow-based reimplementation of the MEDUSA pipeline. It provides a modular and containerized workflow using Nextflow DSL2, with software execution through Docker, Conda or Singularity, which ensures portability, reproducibility, and scalability. The workflow of this pipeline starts with read quality control with FastQC[28], trimming and merging using fastp[53], and optional host decontamination with Bowtie2[10]; MultiQC[54] provides a full report containing visualizations regarding sequence preprocessing. Optionally, clean sequences can be assembled using MEGAHIT[16] with a posterior taxonomic classification carried out by Kaiju[38] or Kraken2[11], while functional annotation relies on a DIAMOND[55]-based alignment to reference databases (NCBi nr by default). It is worthy to mention the flexibility EURYALE offers given its customizable database selection for both taxonomic and functional annotation.

1.6 JAMS[56]

JAMS (Just a Microbiology System) is an integrated framework originally designed to perform the analysis on the NIH's Biowulf system. JAMs is divided into two main modules: JAMSα, which performs single sample analyses, and JAMSβ, which focuses on cross-sample comparisons. JAMSα (the pipeline) integrates tools such as Bowtie2[10] for host removal, MEGAHIT[16] or SPAdes[57] for read assembly, Kraken2[11] for taxonomic classification, and Prokka[58] and InterProScan[59] for gene and protein domain prediction, respectively; JAMSβ uses R-based packages for visualization and statistical analysis. This workflow is executed within Conda environments, and its main advantage relies on the ease to establish comparisons across samples. However, this pipeline does not support binning tools nor genome-quality, and currently, it exhibits restricted deployment flexibility due to optimization for the NIH's Biowulf system, although JAMS is open source and can be installed on any UNIX-based machine.

1.7 MAGNETO[60]

MAGNETO is an automated, modularized and scalable pipeline wrapped with Snakemake[61] and executed with Conda. It is focused on allowing the user the selection of different assembly and/or binning strategies, involving several steps from read pre-processing until MAG annotation and gene catalog generation. The *Pre-processing* module leverages fastp[53], Bowtie2[10] and FastQ Screen[62], whilst the *Assembly* mode uses Simka[63] and hierarchical agglomerative clustering to cluster the samples if the users pre-defines a co-assembly strategy; the reads are assembled using MEGAHIT[16]. Furthermore, contig abundances are computed by alignment against the raw reads to be bin by MetaBAT2[3] afterwards. Quality estimation and dereplication are carried out with CheckM[64] v1.0 and dRep[43], respectively. To end the workflow, a gene catalog is produced for both the contigs and the MAGs by running Prodigal[39], Linclust[65] and CD-HIT[66], and the MAGs are annotated with GTDB-Tk2[25] and eggNOG-mapper[67]. As a special feature, MAGNETO can provide a read-based taxonomy abundance with mOTU[68] profiler. MAGNETO exhibits all the advantages Snakemake wrapping, and executed with Conda, represents such as multi-sample handling, scalability across different computing infrastructures and checkpoint control for workflow restarting.

1.8 MAGO[69]

MAGO is an end-to-end pipeline designed to run over a single execution from a container image (Singularity or Docker); a third option is available as a Virtual Machine (VM). This configuration allows MAGO to offer a streamlined implementation of the entire metagenomics pipeline, including error checking, and computational resource distribution. The tool workflow follows the traditional design with read quality control (fastp[53], FastQC[28]), followed by the assembly step with MEGAHIT[16], metaSPAdes[35] and/or IBDA-UD[70]. MAGO performs binning through multiple algorithms (MetaBAT[71], MaxBin2[5], CONCOCT[4] and BinSanity with multiple configurations). MAG completeness and contamination of MAGs are estimated with CheckM[64]. To conclude the execution, MAGO annotates the MAGs with Prokka[58], and performs taxonomic classification and phylogenetic placement using GTDB-Tk[72]. Moreover, to expand its capabilities, the developers included the possibility of generating phylogenetic trees through ezTree[73], analyzing the pangenome with Roary and measuring ANI with FastANI[74] as an approximation to de-replicate the MAG set.

1.9 metaGEM[75]

metaGEM represents a traditional end-to-end pipeline designed to reconstruct MAGs from metagenomics raw reads; however, its main feature relies on an integrated module that provides genome scale metabolic models (GEMS). The workflow starts with the read quality cleaning using fastp[53] for a subsequent assembly with MEGAHIT[16] and a contig coverage estimation with BWA[76]. The bins are then obtained via three different tools (MetaBAT2[3], MaxBin2[5] and CONCOCT[4]) along a posterior refining by the metaWRAP[23] refinement module. As a result, the bins or MAGs are used as input for CarveMe[77] (Genome Scale Metabolic Models), and SMETANA[78] is called for metabolic interaction predictions and MEMOTE[79] is in charge of generating quality reports. The resulting GEMs can then be used for various downstream analyses, such as predicting metabolic interactions within the community, simulating growth under different conditions, and identifying key metabolic pathways. The pipeline ends with MAG characterization through Prokka[58] and Roary[80] (functional annotation and pangenome analysis), GRiD[81] (growth rate estimation), GTDB-Tk2[25] (taxonomic annotation) and BWA[76] (genome abundance). As additional features, metaGEM identifies eukaryotic MAGs via EukRep[82] and evaluates contamination with EukCC[83]. Also, this pipeline produces taxonomic abundance profiles from the filtered reads using mOTUS2[84]. Naturally, this pipeline exhibits the benefits Snakemake[61] orchestration provides, as mentioned previously.

1.10 MetaGenePipe[85]

MetaGenePipe is a pipeline developed with Workflow Definition Language (WDL), self-executed within a Singularity container, whose primary goal is performing a contig-based functional and taxonomic analysis from short read sequences. It is composed of 4 subworkflows, where the

operation starts with the quality control workflow, the subsequent one assembles the reads with MEGAHIT[16] to map them back against the short reads within the third subworkflow. Meanwhile, the last subworkflow is in charge of gene prediction and functional annotation based on two main strategies: alignment with the Swiss-Prot database and Hidden Markov Models search in KOfam database[86]. Although MetaGenePipe does not include binning software to provide MAGs as main output, its versatility that allows an analysis adapted for eukaryotic and viral analyses with minimal modifications, and its uncommon workflow manager within the pipelines considered in this review, makes MetaGenePipe an interesting alternative for users with advanced computational infrastructures. Additionally, MetaGenePipe is designed to handle a co-assembly strategy in case the user requires this feature.

1.12 Metagenome-Atlas[87]

Metagenome-Atlas is an end-to-end, Snakemake[61]-based and Conda-executed pipeline supporting Illumina short reads and providing a modular workflow. It is divided into four modules, namely Quality Control, Assembly, Genomic Binning and Annotation. The initial module removes host, common contaminants and PCR duplicates, and if necessary, trims low-quality sequences according to user pre-specified parameters. The Assembly module corrects sequence errors based on k-mer coverage, merges paired-end sequences, assembles them using MEGAHIT[16] and/or metaSPAdes[35] along with a contig-length filtering. The following module uses MetaBAT2[3], MaxBin2[5], and optionally VAMB[88] and SemiBin2[21] to bin the contigs; CheckM2[24], BUSCO[89] and GUNC[90] are run to measure the bin quality, as well as DASTool[7] and dRep[43] for bin refinement and MAG dereplication, respectively. For the last module, Metagenome-Atlas taxonomically and functionally annotates the MAGs using GTDB-Tk2[25] and DRAM[91], respectively, and it finally produces a gene catalog through mapping the predicted coding sequences using eggNOG-mapper[67]. Among the main advantages of Metagenome-Atlas, it is possible to describe the possibility of running individual modules and its energetic supporting community and developers. Moreover, the Snakemake wrapper allows for flexibility, multi-sample handling, and adaptability to medium to large projects running on local servers or High-Performance Cluster (HPC) environments.

1.13 Metaphor[92]

Metaphor is a classic metagenomics pipeline aiming at MAG reconstruction and annotation wrapped by Snakemake[61] and leveraging Conda as package manager. The pipeline is triggered by the user with a *.csv* file pointing to the sequence directories and a *.yaml* file with the pipeline configuration. A quality control will be carried out then with FastQC[28] and fastp[53], with a posterior assembly with MEGAHIT[16], contig evaluation with MetaQUAST[93] and mapping against the input sequences using Minimap2[94] and Samtools; the contigs are binned (VAMB[88], MetaBAT2[3], CONCOCT[4]) and refined (DASTool[7]). Metaphor execution finalizes with bin annotation through Prodigal, Diamond, and the NCBI COG database. Complementary to Snakemake orchestration capabilities, Metaphor provides a series of plots depicting runtime and memory with the goal of identifying computational bottlenecks during the analyses.

1.14 MetaWRAP[23]

MetaWRAP is a popular and customizable pipeline built primarily as a command-line framework with a focus on flexibility and user control. MetaWRAP consists of individual modules that can be run independently or combined into custom workflows. Its core functionalities encompasses read QC and cleaning (FastQC[28], Trim Galore and BMTagger), assembly (MEGAHIT[16], metaSPAdes[35], BWA[76] and MetaQUAST[93]), and a binning suite that incorporates MetaBAT2[3], MaxBin2[5], and CONCOCT[4]. MetaWRAP also includes a native refinement module that produces hybrid bin sets to explore over the different variants of each bin (original and hybridized bin sets) to determine the “best bin” according to the user pre-specified quality values based on completeness and contamination (CheckM[64] v1.0). This module is frequently executed in independent metagenomics analysis, and even some pipelines described in this review incorporate it within their workflows. If decided by the user, MetaWRAP offers the possibility of bin re-assembling guided by their previous versions, improving the overall bin quality. For MAG taxonomic and functional analysis, MetaWRAP relies on Prokka[58] and

Taxator-tk[95] (combined with NCBI[31] databases), and it provides visualization modules for summarizing results. Analogous to MAGNETO[60], MetaWRAP can produce read-based taxonomic profiles in parallel. Although MetaWRAP does not integrate full pipeline automation, its high modularity and straightforward design have promoted a wide supporting community. Nonetheless, at the moment of writing this report, MetaWRAP is not maintained by the developers, with the subsequent lack of tool updates.

Nonetheless, given the popularity of MetaWRAP, a Snakemake[61] wrapper was developed to automate the metagenomics analysis known as SnakeWRAP[96]. Therefore, SnakeWRAP can carry out the MetaWRAP end-to-end read processing to generate MAGs in a single run, retaining the flexibility of MetaWRAP while reducing the burden of manual execution and dependency handling. Additionally, SnakeWRAP's integrated environment management via Conda and support for HPC environments enables seamless execution of multiple MetaWRAP modules and samples in parallel, being particularly useful for multi-sample execution.

1.15 MOSHPIT[97]

According to its documentation, *MOSHPIT (MOdular SHotgun metagenome Pipelines with Integrated provenance Tracking) is a toolkit of plugins for whole metagenome assembly, annotation, and analysis built on the microbiome multi-omics data science framework QIIME 2[98]. MOSHPIT enables flexible, modular, fully reproducible workflows for read-based or assembly-based analysis of metagenome data*. The core components of MOSHPIT include q2-assembly, which provides functionalities for genome assembly and quality control, and q2-annotate, which supports contig binning, taxonomic classification, and functional annotation. Additional plugins, such as q2-viromics and q2-amrfinderplus, extend capabilities to viral sequence detection and antimicrobial resistance gene annotation, respectively. In technical terms, MOSHPIT must be run locally or on an HPC environment with the possibility to execute the processes in parallel by the explicit declaration of partitions, a native QIIME2 functionality. Further, the entire QIIME2 ecosystem relies on Conda, and hence this a *sine-qua-non* requisite to perform MAG reconstruction with MOSHPIT.

1.16 nIMP3[99]

nIMP3 is a Nextflow-based reimplementation of the IMP (Integrated Meta-omic Pipeline) workflow that assembles metagenomics (MG) and metatranscriptomics (MT) datasets together. nIMP3 handles preprocessed and contaminant-free MT and MG reads (FastQC[28], SortMeRNA[100], BBTools[101]), and jointly assembles them in a *hybrid* and iterative process using MEGAHIT[16]. Additionally, nIMP3 performs taxonomic profiling with mOTUs[102] and Kraken2[11], as well as functional profiling with gffquant[103]. Unlike the original IMP pipeline, nMP3 does not include a binning module, and thus it cannot recover MAGs. Nonetheless, nIMP3 offers a lighter, reproducible, and integrative pipeline for multi-omics metagenome/metatranscriptome processing.

1.17 SnakeMAGs[104]

SnakeMAGs is a simple yet useful pipeline that as its name indicates is controlled by a Snakemake[61] wrapper with Conda as software administrator. It integrates basic modules starting with quality control with Illumina-utils[105] and Trimmomatic[27], and if required, host removal with Bowtie2[10]. Afterwards, the reads are assembled through MEGAHIT[16], the contigs are binned by MetaBAT2[3], a quality assessment is carried out with CheckM[64] v1.1 and GUNC[90], MAG abundances are obtained using CoverM[106], and finally the taxonomic classification is performed using GTDB-Tk2[25]. Similar to the previous pipelines governed by Snakemake, SnakeMAGs eases automation, reproducibility, scalability and workflow management.

1.18 SPIRE[107]

The SPIRE project employs a Nextflow-based pipeline that has been used to process and annotate more than 100,000 metagenomes belonging to more than 700 studies. The workflow incorporates tools such as NGLess[108] for read trimming and decontamination, MEGAHIT[16] for

assembly, Prodigal[39] for gene prediction and barrnap[109] for ribosomal RNA detection. Moreover, contig binning is carried out with MetaBAT2[3] with a complementary genome quality assessment using CheckM2[24] and GUNC[90], and the workflow ends with taxonomic classification (GTDB-Tk2[25]) and functional annotation (eggNOG-mapper[67], abricate[110], RGI[50] and Macrel[111]). Among the advantages SPIRE offers, the possibility to perform antimicrobial resistance gene prediction and the annotation of virulence factors stand out, as well as its scalability, reproducibility across high-performance and cloud environments, and standardized processing, enabling consistent comparisons across global datasets. Nonetheless, at the moment of writing this report, this pipeline is aiming to be executed at online platforms like CloWM[112] as it is lacking defined environments or container images, and the input data should be already hosted at the sequencing archives such as ENA, DDBJ or SRA.

1.19 Sunbeam[113]

Sunbeam is a modular pipeline orchestrated by Snakemake[61] with Conda as dependency manager; this configuration makes Sunbeam analysis reliable, reproducible and scalable. The main feature Sunbeam depicts is its modularized and extensible design that allows users to build off the core functionality. The execution backbone of Sunbeam is represented by an initial quality control that encloses adapter trimming, host read removal and low-complexity filtering (Trimmomatic[27], FastQC[28], BWA[76] and Komplexity), followed the assembly of reads into contigs with MEGAHIT[16] along with their corresponding annotation with Prodigal[39], BLAST[37] and Diamond[55] (with nucleotide or protein databases). As complementary procedures, Sunbeam maps the reads to reference genomes (user pre-specified) and delivers a taxonomic assignment of the clean reads using Kraken[114] v1.0. As previously stated, its modularization and ready-to-use templates to create new modules have enabled the development of additional extensions for assigning metagenomic reads to a full bacterial phylogeny, single genome assembly, among others.

2. Long-read focused pipelines

2.1 EasyNanoMeta[115]

EasyNanoMeta is a specialized pipeline designed to process ONT long reads either solely or in combination with short reads (hybrid assembly). This pipeline relies on a dual approach that uses both assembly-based and assembly-free strategies. Particularly, EasyNanoMeta incorporates four assemblers (metaFlye[116], OPERA-MS[117], metaSPAdes[35], MetaPlatanus[118]), five binners (SemiBin2[21], MetaBAT2[3], MaxBin2[5], CONCOCT[4], VAMB[88]) and a polishing tool (NextPolish[119]) to assure the best possible outcome. Additionally, once the bins are obtained, it performs the common tasks such as functional annotation with Prokka[58], quality control with CheckM2[24], phylogeny inference with PhyloPhlan[120] and taxonomic classification with GTDB-Tk2[25]. For the assembly-free methodology, EasyNanoMeta provides a full report containing composition, diversity and correlation among the identified species with Kraken2[11] and Centrifuge[121]. Regarding operational characteristics, this pipeline can be run automatically on a Singularity/Apptainer image that streamlines the setup process and minimizes dependency issues or experienced users can execute individual modules through shell scripts that rely on Conda environments.

2.2 Hi-Fi-MAG-Pipeline[122]

Hi-Fi-MAG is a simple, yet time-saving pipeline developed and maintained by Pacific Biosciences specially designed to build MAGs from Hi-Fi reads (long PacBio reads). It encompasses different binning tools (MetaBAT2[3] and SemiBin2[21]) along with DASTool[7] as refinement software; CheckM2[24] serves a quality control tool, where contigs above 500 kb are kept as single bins if they show a completeness above 93%, otherwise they are sent back to the binning module. This approach enhances the recovery of high-quality and single-contig MAGs, outperforming traditional binning methods. After MAG de-replication, taxonomic annotation is achieved with GTDB-Tk2[25], and a complete graphical report is compiled automatically. One important caveat about this workflow is represented by its lack of assembly step, and hence the user must prepare the assembly of the PacBio sequences beforehand using tools such as hifiasm[123] in its meta version, metaFlye[116], OPERA-MS[117],

among others. Hi-Fi-MAG-Pipeline requires Conda as software manager, and it is orchestrated by Snakemake[61].

2.3 Mapler[124]

Mapler is a pipeline specifically designed to handle PacBio HiFi long reads. Mapler workflow is orchestrated by Snakemake along with Conda for package management, enabling scalable execution on local or cluster systems. Regarding the specific tools encompassed by Mapler, state-of-the-art assemblers such as metaMDBG[125], hifiasm-meta[123], metaFlye[116] and OPERA-MS[117] are available, with MetaBAT2[3] as the binning tool. Later on the workflow, each bin is classified taxonomically via GTDB-Tk2[25] or Kraken2[11], and genome quality is evaluated using CheckM2[24] standards. Mapler aligns reads back to contigs with Minimap2[94] to compute novel metrics including the aligned read percentage and aligned base percentage, stratified across quality categories. It is important to mention that Mapler accepts assemblies and bins as input to skip part of the process, and it includes a parallel analysis, where assembled versus unassembled reads are contrasted by evaluating k-mer distributions (KAT[126]), read quality (FastQC[28]), and taxonomic composition (Kraken2 + Krona[41]). As a result, by combining classic bin-based metrics with read-to-contig alignment statistics, Mapler assists in estimating how much of the sequence diversity remains uncaptured.

2.4 NanoPhase[127]

NanoPhase is a pipeline that enables building high-quality MAGs from ONT long reads, optionally enhanced with short read-based MAG polishing. The backbone of the pipeline is represented by an assembly with metaFlye[116] followed by contig binning with MetaBAT2[3] and MaxBin2[5], and bin refinement with a MetaWRAP[23] module. To estimate abundance and coverage, the contigs are mapped against the reads, and several polishing rounds with Racon[128] and medaka, complete the workflow to generate high-accuracy final bins; If the user decides to include short reads in the analysis, these are used for polishing with Pilon[129]. Complementary, MetaQuast[93] and CheckM[64] v1.0 are in charge of MAG quality control, IDEEL[130] evaluates the fraction of predicted full-length proteins in each MAG, full-length proteins are detected via alignment with UniProtKB[131], and Prokka[58] serves as functional annotation software. Remarkably, NanoPhase allows prophage and active prophage identification within the reconstructed MAGs with VIBRANT[132] and PropagAtE[133]. Among pipeline technical specifications, this pipeline requires Conda as package manager and it offers parallelized execution with GNU Parallel to speed up the analysis.

3. Dual pipelines

3.1 GEN-ERA[134]

GEN-ERA suite is a collection of Nextflow[9] pipelines aiming at supporting MAG reconstruction and annotation with as many methodologies as possible starting from either short or long reads. Specifically, this toolbox counts with more that 10 workflows specifically designed for tasks ranging from assembly and binning, quality assessment and decontamination, orthologous inference and maximum likelihood phylogenomic analyses, SSU rRNA phylogeny (constrained by ribosomal phylogenomic), Average Nucleotide Identity (ANI) clustering, taxonomic identification and metabolic modelling. Moreover, GEN-ERA incorporates specific tools designed to handle eukaryotic assembly annotation such as BRAKER2[135] and AMAW[136]. Thus, GEN-ERA suits almost all requirements any user might demand given the variety of goals that can be achieved within a single software suite. From a technical point of view, operational GEN-ERA features, Nextflow-managed and Singularity-executed, ensures portability and reproducibility across environments.

3.2 Metagenomics-Toolkit[137]

Metagenomics-Toolkit is a workflow designed to increase scalability of task execution, enabling optimal resource allocation from its machine learning-optimized assembly step. This optimized assembly tailors the peak RAM value requested by a metagenome assembler to match actual requirements, thereby minimizing the dependency on dedicated high-memory hardware.

Metagenomics-Toolkit is wrapped by Nextflow[9] and powered with Docker containerization technology, and it can take either short or Oxford Nanopore (ONT) long reads as input. As a result, this pipeline is highly scalable and adaptable across computational infrastructures with a backbone workflow that relies on the traditional MAG-aimed steps such as quality control, assembly, binning, and annotation, plus an aggregation module that captures the output from each sample to "polish" the final MAGs. Regarding special features offered by Metagenomics-Toolkit, it offers plasmid identification based on various tools, the recovery of unassembled microbial community members, and the discovery of microbial interdependencies through a combination of dereplication, co-occurrence, and genome-scale metabolic modeling.

3.3 metagWGS[138]

metagWGS is one of the most recently released pipelines whose main differential is related with the possibility to assemble either short reads or long sequences (PacBio). This Nextflow[9] pipeline is built off Singularity with consequent benefits this kind of setup brings as discussed previously. It incorporates a wide variety of tools as it must ensure a proper workflow for both types of sequencing technologies in a traditional end-to-end framework divided into 8 steps. The first step aims at cleaning and performing quality control with proper tools according to the input, while the second step allows the assembly of the sequences using either metaSPAdes[35]/MEGAHIT[16] for short sequences and hifiasm[123]/metaFlye[116] for PacBio reads. Following with the process, this pipeline filters the contigs and performs structural annotation during steps 3 and 4, respectively; step 5 is designed to estimate contig abundance by mapping them against the reads. Afterwards, a complete subworkflow for functional annotation is undergone with eggNOG-mapper[67] at its core (step 6), and contig taxonomic affiliation is achieved through *home-made* scripts (step 7) to conclude with step 8, where the contigs are binned with MaxBin2[5], MetaBAT2[3] and CONCOCT[4]. Furthermore, metagWGS utilizes Binette[139], a state-of-the-art binning refinement tool designed to construct high-quality MAGs from the output of multiple binning tools. As a special remark, metagWGS performs read taxonomic profiling via Kaiju, as well as contig annotation that includes an in-house algorithm and mapping against the reads.

3.4 MG-TK[140]

MG-TK (Metagenomic Toolkit) performs read assembly (SPAdes[57], MEGAHIT[16], Flye[141], metaMDBG[125]) and binning (MetaBAT2[3], SemiBin2[21], MetaDecoder[142]), gene prediction, and clustering into nonredundant gene catalogs, followed by abundance estimation and functional annotation. It is structured around three main phases: processing raw sequences, building a gene catalog, and reconstructing species from MAGs with downstream phylogenetic analyses. It produces a wide range of outputs, including assemblies, MAGs, gene predictions, SNP calls and mapping outputs. A special remark MG-TK exhibits is its ability to generate detailed abundance matrices for both taxonomic and functional features, with hierarchical summaries available at multiple levels. The taxonomic profiles are reported using GTDB[143] lineages, while functional annotations are provided for major databases such as KEGG[144], SEED[145], CAZy[146], eggNOG[45], and TCDB[147]. MG-TK also estimates completeness of functional modules, such as KEGG pathways, and links genes to multiple annotations for deeper exploration by the user. Beyond gene catalogs, MG-TK integrates MAG/MGS (Metagenomics Species) information, associating MAGs with their metagenomic species and providing detailed gene content, including representative MAGs for each species. Additionally, MG-TK can provide assembly-independent profiles via a wide variety of tools including riboFinder[148], MetaPhlAn[47] and mOTUs[102].

3.5 VEBA[149]

VEBA (Viral Eukaryotic Bacterial Archaeal) is a Conda-executed pipeline designed that enables the recovery and classification of genomes from all domains of life including archaeas, prokaryotes, microeukaryotes, and viruses. It starts with a common short read-preprocessing and assembly from which the process is bifurcated for prokaryotic and viral binning; unbinned contigs from the viral module are reincorporated into the prokaryotic contig set. Residual contigs from the prokaryotic module are then considered for eukaryotic MAG generation to proceed with the annotation and classification covering the genomes obtained in each module. Hence, several databases are

considered at this step such as UniRef50/90[150], MIBiG[151], VFDB[49], CAZy[146], KOfamKOALA[86], Pfam[152], NCBIfam-AMR[153] and AntiFam[154]. Also, a joint phylogeny is obtained based on MAG-gene models and lineage marker detection. An interesting approach VEBA follows is represented by the module *coverage.py* that collects all the unbinned contigs, from viral, eukaryotic and prokaryotic steps, to pursue a pseudo-coassembly, where iteratively the reference fasta (built from the contigs) and the sorted BAM files used as a final pass through prokaryotic and eukaryotic binning modules. This pseudo-coassembly approach is optional, being easily enabled during the workflow execution; the pipeline documentation widely discusses when this type of assembly should be used in specific cases. Notably, VEBA automates the detection of candidate phyla radiation (CPR) bacteria and integrates a consensus microeukaryotic database to optimize gene modeling and taxonomic classification.

4. Hybrid pipelines

4.1 Aviary[155]

Aviary is a modular, Snakemake[61]-based pipeline, with Conda as package manager, designed for single or hybrid metagenomic assembly and MAG recovery, supporting both short and long-read input sequences. The workflow is distributed in 8 modules following a traditional workflow starting with quality and diversity assessment of the reads, followed by a discriminated assembly according to the type of input, MEGAHIT[16] or metaSPAdes[35] for short reads only or metaFlye[116] in case of long reads solely. For hybrid assembly the process is divided into four stages: polishing with Racon[128] and Pilon[129], metrics-based filtering, assembly and discard of low-quality bins and re-assembly with Unicycler[156]. The pipeline proceeds with a subsequent assembly evaluation in terms of fragmentation, misassembly detection and diversity quantification, and a complementary module moves forward with a read mapping of the assembly and abundance statistics calculation. To continue with the workflow, the contigs are binned using up to 6 tools (MetaBAT2[3], Rosella[157], MetaBAT1[71], VAMB[88], MaxBin2[5] and CONCOCT[4]) and refined afterwards with 5-time loop that includes CheckM2[24], Rosella Refine and DASTool[7]. The pipeline ends with MAG recovery assessment via CoverM[106], CheckM2 and SingleM to proceed with MAG annotation through GTDB-Tk2[25], Prodigal[39] and eggNOG[45]. Variant calling, ANI analysis and genotype recovery with Lorikeet[158] are interesting attributes offered by Aviary as a complement to the traditional genomic feature detection. Aviary's design presents a series of advantages that include the possibility of running modules, multi-sample handling and scalability across different computational infrastructures.

4.2 MUFFIN[159]

MUFFIN is a reproducible pipeline built with Nextflow[9] designed for hybrid assembly by integrating short-read (Illumina) and long-read (nanopore) sequencing data. MUFFIN begins its workflow with a quality control of the reads (fastp[53] and Filtlong) to progress through hybrid assembly (metaSPAdes[35] or metaFlye[116] with polishing) and differential binning (CONCOCT[4], MetaBAT2[3], and MaxBin2[5]). After bin refining with the MetaWRAP[23] refinement module, a hybrid reassembly is pursued with Unicycler[156]. The pipeline ends with bin classification through CheckM[64] v1.1 and sourmash[13] (combined with GTDB[143]), and with bin annotation with eggNOG[45] and a KEGG[144] parser, providing high-quality, annotated MAGs and insights into the metabolic potential of the microbial community. Optionally, the user can provide metatranscriptomics data to perform a de novo transcript assembly (Trinity[160]), quantification (Salmon[161]) and annotation (eggNOG). Additionally, given its modularity design, the workflow can start as well with user-provided bins, differential reads or only RNA-seq data. MUFFIN can be executed with either Conda or Docker, and its native Nextflow features confer to it the possibility to restart the pipeline in case of failing, run on different computing infrastructures, multi-sample handling, among others.

4.3 nf-core/mag[162]

nf-core/mag is a Nextflow[9] pipeline developed following the nf-core guidelines that ensures robustness and reproducibility. It supports both short-read and long-read sequences, as well as hybrid datasets, and it leverages a modular design, containerization (Docker, Singularity, among others) and

package managers (Conda) to confer portability across different computing environments, including HPC and cloud systems. Beyond these important features, as part of the workflow orchestration, nf-core/mag can handle multi-sample input, it can be restarted if it is interrupted at any point thanks to its native checkpoint control and different assembly/binning modes can be selected. This pipeline encompasses tools for quality control of the reads (Porechop[163], Filtlong[164], NanoPack2[165], fastp[53]), host removal (Bowtie2[10]), adapter trimming (AdapterRemoval[166]), and several assemblers (MEGAHIT[16], metaSPAdes[35], Flye[141], metaMDBG[125], hybridSPAdes[167]). In addition, it offers three binning software options (MetaBAT2[3], MaxBin2[5] and CONCOCT[4]) along with an optional refinement tool (DASTool[7]). nf-core/mag checks assembly and bin quality through several tools that include CheckM2[24], MetaQUAST[93], BUSCO[89] and GUNC[90], and for genome annotation, it uses GTDB-Tk2[25] or CAT[168] (taxonomic) and Prokka[58] or MetaEuk[169] (functional). As special features, this pipeline can carry out a taxonomic annotation of the sequences (Kraken2[11] and Centrifuge[121]), validates the presence of typical ancient DNA damages (PyDamage[170]), attempts MAG domain classification with Tiara[171] and identifies viruses after assembly with geNomad[172]. After workflow execution, nf-core/mag generates detailed multi-sample summaries through MultiQC[54], and it creates HTML reports to track resource usage. Finally, the nf-core framework is actively maintained and updated as it relies on a numerous and enthusiastic developing community.

4.4 ngs-preprocess-MpGAp-Bacannot[173]

Ngs-preprocess, MpGAP and Bacannot are a series of Nextflow[9]-based and container-powered pipelines designed to achieve a wide variety of specific tasks. ngs-preprocess performs several quality-control steps required for Next-Generation Sequencing (NGS) data assessment, while MPGAP supports de novo genome assembly from Illumina, PacBio, and ONT reads, enabling short-read, long-read, and hybrid assemblies using tools like metaSPAdes[35], metaFlye[116], Canu[174], and Unicycler[156], followed by polishing and quality assessment. Meanwhile, Bacannot provides an annotation workflow that incorporates gene prediction, rRNA detection, sequence typing, KEGG-based metabolic reconstruction, and secondary metabolite identification, integrating tools such as Prokka[58], Bakta[175], Barrnap[109], MLST[176], KofamScan[86], KEGGDecoder[177], and antiSMASH[178]. As an additional analytical procedure, Bacannot incorporates additional support for methylation analysis via Nanopolish[179]. Noticeably, this set of pipelines do not include at any point neither contig binning nor bin quality assessment; however, the smooth interconnection among the pipelines makes them an interesting option for metagenome assembly and annotation, boosted by the native benefits conferred by Nextflow and container technology.

4.5 SqueezeMeta[180]

SqueezeMeta is a fully automatic pipeline written in Perl scripts that relies on Conda for software execution. As special features, this pipeline can handle short and long reads (ONT and Hi-Fi) in both single or hybrid approaches, supports for de-novo metatranscriptome assembly and hybrid metagenomics/metatranscriptomics analysis, carries out taxonomic annotation of unassembled reads, and empowers the user with a GUI application for downstream analysis. Also, SqueezeMeta's flexibility enables different assembly modes such as *sequential* (samples assembled individually), *co-assembly* (samples assembled ensemble), *merged* (samples assembled individually with a posterior pooling) and *seqmerge* (similar to merged with a guided pooling based on assembly similarity). This pipeline follows the traditional workflow by applying quality filtering and trimming with Trimmomatic[27], then the reads are assembled by MEGAHIT[16] and SPAdes (rnaSPAdes[181], Canu[174] and metaFlye[116] are run if transcriptomics or long read data are provided) to be binned afterwards with MaxBin2[5], MetaBAT2[3] and CONCOCT[4]; DASTool[7] is in charge of bin refinement. MAG Quality checks are established through CheckM2[24], and optionally taxonomic classification is achieved by GTDB-Tk2[25]. To complement MAG annotation with KEGG[144] and MetaCyc[182], SqueezeMeta analyzes the assembly by performing an homology searching against taxonomic and functional databases, an HMMER search against Pfam[152] database, and an estimation of taxa and function abundances. An important remark of this pipeline is its numerous and helpful developing and maintaining community.

5. Web-based pipelines with external computational resource support

5.1 BV-BRC[183]

BV-BRC (Bacterial and Viral Bioinformatics Resource Center) is web-based platform that supports a broad spectrum of microbial genomics analyses, including genome-resolved metagenomics. This platform offers an intuitive interface to perform tailored quality control, assembly, binning, annotation, and downstream comparative analyses. For MAG building, BV-BRC has developed a specific metagenomic binning service, which offers genome assembly with metaSPAdes[35] or MEGAHIT[16] and a customized approach for genome binning based on kmer distribution and multi-genome functionality. Moreover, BV-BRC leverages PATRIC[184] genomes to create reference bins as a starting point for annotation with RASTtk[185] and/or VIGOR4[186]. Regarding technical features, BV-BRC runs entirely on a remote infrastructure, allowing users to execute workflows without local installations or advanced computational setups. Aside from the features already mentioned, customizable analysis jobs, visualization tools and integrated comparative genomics tools are available, making BV-BRC a valuable resource for users seeking an accessible, reproducible, and data-rich environment for metagenomic studies.

5.2 Galaxy[187]

Galaxy is a web-based platform and open-source project that empowers scientists all over the world to conduct bioinformatics analysis in an user-friendly and intuitive graphical interface that requires no programming skills. Galaxy offers a broad range of tools covering genomics, transcriptomics, metagenomics, among many others, where the user is free to select the software that best suits their needs. In addition, the users can share their workflows in the platform, and therefore users can just follow pre-established methodologies validated by a world-wide community. As a result, there are multiple pipelines designed for MAG reconstruction that feature common tools like MEGAHIT[16] for assembly, MetaBAT2[3] or MaxBin2[5] for binning, and Prokka[58] or GTDB-Tk2[25] for annotation and classification. Also, given Galaxy's flexibility the traditional workflow can be expanded to include long reads, accomplish read-based taxonomic profiling or detect and classify viral sequences. Being so, Galaxy ensures reproducibility through automatic tracking of parameters and tool versions, and supports HPC and cloud deployment, making it scalable for projects of various sizes. Notwithstanding, the users may experience limitations in performance for large datasets and/or delays in result processing as Galaxy's community of users grows every day with the subsequent demand for more computational resources.

5.3 IDseq[188]

IDseq is an open-source, cloud-based platform developed for metagenomic next-generation sequencing (mNGS) analysis. IDseq has a specific scope focused on pathogen detection, antibiotic resistance detection and infection control. IDseq supports short-reads or long reads (ONT) to provide analyses that encompass host read removal, quality control, alignment, and taxonomic classification using a curated reference database based on NCBI[31] nt and nr databases. Although IDseq is not primarily focused on MAG reconstruction, it is highly valuable in the initial stages of metagenomics data analysis projects. As interesting remarks, IDseq's results are visualized through interactive dashboards that provide taxonomic trees, abundance plots, and detailed sample metrics thanks to its web-based interface that requires minimal bioinformatics expertise. Also, the users can find alternative pipelines for viral consensus genome recovery and antimicrobial resistance gene detection.

5.4 IMG/M[189]

IMG/M (Integrated Microbial Genomes & Microbiomes) developed by the DOE (the United States Department Of Energy) Joint Genome Institute for the annotation and comparative analysis of microbial genomes and metagenomes. IMG/M is designed primarily to host and annotate genomes, offering a pipeline, running on their servers, that takes contigs to bin them via SemiBin2[21], with subsequent quality control by CheckM[64]. The taxonomic annotation is given by GTDB-Tk[72], and functional annotation is supported using resources such as KEGG[190], COGs[191], Pfam[152] and

TIGRFAMs[192], enabling pathway reconstruction and metabolic profiling; as inferred from this workflow description, the users need to perform the assemble step elsewhere. This platform also incorporates comparative tools to allow exploration of gene content, pathway coverage, phylogenetic profiles, and functional similarities across datasets. It is important to mention that datasets submitted to IMG/M are initially private but must eventually become public. IMG/M enforces an embargo period, after which annotated data are released and cannot be withdrawn, although updates are allowed.

5.4 KBase[193]

KBase (the United States Department Of Energy Systems Biology Knowledgebase) is a collaborative, web-based platform that enables researchers to perform comprehensive metagenomics analyses through its customized interactive Narrative Interface. This platform allows users to build and share workflows (narratives) for genome assembly, comparative genomics, metagenomics, among others. Specifically, the metagenomics narrative offers running MAG-centered pipeline steps such as quality control, assembly (e.g., metaSPAdes[35], MEGAHIT[16]), binning (i.e., MetaBAT2[3]), annotation (e.g., RASTtk[185], DRAM[91]), and metabolic modeling using ModelSEED[194]. KBase platform offers automated data provenance, seamless integration with public databases, and interactive visualizations to interpret MAG quality, taxonomy, and metabolic pathways. The possibility of running analyses using external resources makes KBase a powerful and accessible environment for genome-resolved metagenomics, particularly valuable for users lacking access to HPC systems.

5.5 MGnify[195]

MGnify is a web-based platform hosted by EMBL-EBI with an automatized service for submitting and annotating microbiome-derived sequence data. It counts with a standardized pipeline that receives raw reads to perform functional and taxonomic annotation with an extensive series of tools encompassing mOTUs2[84], InterProScan[59], KEGG annotation (hmmscan[196]), eggNOG-mapper[67] and/or antiSMASH[178]. Optionally, MGnify offers the possibility for read assembly through metaSPAdes with a prior contamination removal to continue with the annotation. In the recent years, MGnify has evolved to accept and process long reads from PacBio and ONT with the pipeline MGnify-lr that carries out read pre-filtering, assembly with Flye and re-mapping against the initial sequences. Furthermore, users can contribute to the resource MGnify Genomes which stores a genome catalogues each user can create with their own MAGs. Once the MAGs are submitted to this space, they are automatically analyzed with a pipeline that establishes overall quality and annotates them. Given that MGnify is a service controlled by EMBL-EBI, the user is only requested to submit the data and make it publicly available before the analysis to ENA. As a result, MGnify is a powerful computational resource and user-friendly as the user interacts with the platform to upload the data through its web interface, taking the burden off the user. However, MGnify's reliance on predefined workflows may limit flexibility for users seeking to customize specific steps or parameters in the analysis, while at the same time heavy use by multiple users may delay result delivery.

5.6 WGSA2+/LoRA[197]

The Nephele suite offers two independent metagenomics analysis pipelines: WGSA2+ for short-read data and LoRA to handle PacBio or ONT reads. Briefly, WGSA2+ performs quality control and host removal using tools such as fastp[53] and Kraken2[11], assembles reads with metaSPAdes[35], and optionally bins contigs into MAGs with MetaBAT2[3], assessing MAG quality with CheckM[64]. Taxonomic classification is achieved through Kraken2, whilst eggNOG-mapper[67] is in charge of functional annotation. LoRA, on its side, uses metaFlye[116] for assembly, integrates the same binning and functional annotation tools, and expands the classification module with inclusion of GTDB-Tk2[25] and CheckM2[24]. Both pipelines can generate taxonomic profiles, functional summaries and detect antibiotic resistance genes through Nephele’s user-friendly cloud interface; WGSA2+ supports metatranscriptome assembly from RNA-seq data. As a result, WGSA2+/LoRA represent a great option for users who are experienced at command line tool execution or with limited local computing resources. However, Nephele’s platform usage is limited as it relies on AWS for software execution, and therefore users receive a fixed number of *use codes*, and in case of intensive resource demands, they can request extended access.

6. Special pipelines

6.1 Pipeline for ancient DNA[198]

MAG recovery from ancient DNA can be challenging due to DNA intrinsic properties such as degradation, fragmentation, chemical damage, low-abundance and contamination. Nonetheless, a validated pipeline to manage this type of data is proposed by Standeven *et al.* (2024)[198], where the MAGs are obtained by following the classic steps involving quality check, decontamination, assembly, binning, bin quality assessment and refinement, and taxonomic annotation. The main advantage of this pipeline is the integration of different bin software, and it can also authenticate the sequence provenance by estimating damage authentication of the host DNA (mainly human) via mapDamage2[199]. Despite its validation to recover high-quality MAGs, this pipeline is only proposed, and it has not been properly compiled in a single repository or container, and hence users should run the tools manually or leverage any of the other available pipelines in this suite.

6.2 Eukfinder[200]

Eukfinder is a specialized pipeline designed to recover microbial eukaryotic genomes, including both nuclear and mitochondrial DNA. Considering the inherent complexity and underrepresentation of eukaryotic genomes in metagenomics, this tool is composed by two workflows: the first one for Illumina short reads (Eukfinder_short) and another one for assembled contigs or long-read data (Eukfinder_long). In the workflow for short reads, they are first classified into five major taxonomic groups using Centrifuge[121] and PLAST[201], and afterwards 'Eukaryotic' and 'Unknown' reads are subsequently assembled and reclassified to refine candidate eukaryotic sequences. On the other hand, the long-read version focuses on classifying pre-assembled contigs before proceeding to genome binning and downstream analysis. The binning procedure is common to both approaches and it relies on MyCC[202] output, Centrifuge[121], and PLAST results in customized and tailored integration of kmer analysis and contigs mapping to eukaryotic genomes. Given its specificity, Eukfinder represents a flexible solution for studying eukaryotic microbial communities in environmental metagenomics.

References

1. Eren, A. M. *et al.* Community-led, integrated, reproducible multi-omics with anvi'o. *Nat. Microbiol.* **6**, 3–6 (2020).
2. Breitwieser, F. P., Baker, D. N. & Salzberg, S. L. KrakenUniq: confident and fast metagenomics classification using unique k-mer counts. *Genome Biol.* **19**, 198 (2018).
3. Kang, D. D. *et al.* MetaBAT 2: An adaptive binning algorithm for robust and efficient genome reconstruction from metagenome assemblies. *PeerJ* **2019**, (2019).
4. Alneberg, J. *et al.* Binning metagenomic contigs by coverage and composition. *Nat. Methods* **11**, 1144–1146 (2014).
5. Wu, Y. W., Simmons, B. A. & Singer, S. W. MaxBin 2.0: an automated binning algorithm to recover genomes from multiple metagenomic datasets. *Bioinformatics* **32**, 605–607 (2016).
6. Graham, E. D., Heidelberg, J. F. & Tully, B. J. BinSanity: unsupervised clustering of environmental microbial assemblies using coverage and affinity propagation. *PeerJ* **5**, e3035 (2017).
7. Sieber, C. M. K. *et al.* Recovery of genomes from metagenomes via a dereplication, aggregation and scoring strategy. *Nat. Microbiol.* **3**, 836–843 (2018).
8. Fuentes-Santander, F., Curiqueo, C., Araos, R. & Ugalde, J. A. BugBuster: a novel automatic and reproducible workflow for metagenomic data analysis. *Bioinforma. Adv.* **5**, vbaf152 (2025).
9. Tommaso, P. D. *et al.* Nextflow enables reproducible computational workflows. *Nat. Biotechnol.* **35**, 316–319 (2017).
10. Langmead, B. & Salzberg, S. L. Fast gapped-read alignment with Bowtie 2. *Nat. Methods* **9**, 357–359 (2012).
11. Wood, D. E., Lu, J. & Langmead, B. Improved metagenomic analysis with Kraken 2. *Genome Biol.* **20**, 1–13 (2019).
12. Lu, J., Breitwieser, F. P., Thielen, P. & Salzberg, S. L. Bracken: Estimating species abundance in metagenomics data. *PeerJ Comput. Sci.* **2017**, e104 (2017).
13. Irber, L. *et al.* sourmash v4: A multitool to quickly search, compare, and analyze genomic and metagenomic data sets. *J. Open Source Softw.* **9**, 6830 (2024).
14. Prosperi, M. & Marini, S. KARGA: Multi-platform Toolkit for k-mer-based Antibiotic Resistance Gene Analysis of High-throughput Sequencing Data. in *2021 IEEE EMBS International Conference on Biomedical and Health Informatics (BHI)* 1–4 (2021).
15. Marini, S., Boucher, C., Noyes, N. & Prosperi, M. The K-mer antibiotic resistance gene variant analyzer (KARGVA). *Front. Microbiol.* **14**, (2023).
16. Li, D. *et al.* MEGAHIT v1.0: A fast and scalable metagenome assembler driven by advanced methodologies and community practices. *Methods* **102**, 3–11 (2016).
17. Altschul, S. F., Gish, W., Miller, W., Myers, E. W. & Lipman, D. J. Basic local alignment search tool. *J. Mol. Biol.* **215**, 403–410 (1990).
18. Laetsch, D. R. & Blaxter, M. L. BlobTools:

Interrogation of genome assemblies. Preprint at https://doi.org/10.12688/f1000research.12232.1 (2017).
19. Arango-Argoty, G. *et al.* DeepARG: A deep learning approach for predicting antibiotic resistance genes from metagenomic data. *Microbiome* **6**, 1–15 (2018).
20. Figueroa III, J. L., Dhungel, E., Bellanger, M., Brouwer, C. R. & White III, R. A. MetaCerberus: distributed highly parallelized HMM-based processing for robust functional annotation across the tree of life. *Bioinformatics* **40**, btae119 (2024).
21. Pan, S., Zhao, X. M. & Coelho, L. P. SemiBin2: self-supervised contrastive learning leads to better MAGs for short- and long-read sequencing. *Bioinformatics* **39**, i21–i29 (2023).
22. Wang, Z. *et al.* Effective binning of metagenomic contigs using contrastive multi-view representation learning. *Nat. Commun.* **15**, 1–14 (2024).
23. Uritskiy, G. V., Diruggiero, J. & Taylor, J. MetaWRAP - A flexible pipeline for genome-resolved metagenomic data analysis. *Microbiome* **6**, 1–13 (2018).
24. Chklovski, A., Parks, D. H., Woodcroft, B. J. & Tyson, G. W. CheckM2: a rapid, scalable and accurate tool for assessing microbial genome quality using machine learning. *Nat. Methods* **20**, 1203–1212 (2023).
25. Chaumeil, P.-A., Mussig, A. J., Hugenholtz, P. & Parks, D. H. GTDB-Tk v2: memory friendly classification with the genome taxonomy database. *Bioinformatics* **38**, 5315–5316 (2022).
26. Benavides, A., Sanchez, F., Alzate, J. F. & Cabarcas, F. DATMA: Distributed Automatic Metagenomic Assembly and annotation framework. *PeerJ* **8**, (2020).
27. Bolger, A. M., Lohse, M. & Usadel, B. Trimmomatic: a flexible trimmer for Illumina sequence data. *Bioinformatics* **30**, 2114–2120 (2014).
28. Simon, A. FastQC A Quality Control tool for High Throughput Sequence Data. FastQC [Online]. Available online at: http://www.bioinformatics.babraham.ac.uk/projects/fastqc/ (2010).
29. Magoč, T. & Salzberg, S. L. FLASH: fast length adjustment of short reads to improve genome assemblies. *Bioinformatics* **27**, 2957–2963 (2011).
30. Ontiveros-Palacios, N. *et al.* Rfam 15: RNA families database in 2025. *Nucleic Acids Res.* **53**, D258–D267 (2025).
31. Goldfarb, T. *et al.* NCBI RefSeq: reference sequence standards through 25 years of curation and annotation. *Nucleic Acids Res.* **53**, D243–D257 (2025).
32. Maidak, B. L. *et al.* The RDP (Ribosomal Database Project). *Nucleic Acids Res.* **25**, 109–110 (1997).
33. Quast, C. *et al.* The SILVA ribosomal RNA gene database project: improved data processing and web-based tools. *Nucleic Acids Res.* **41**, D590–D596 (2013).
34. Benavides, A., Isaza, J. P., Niño-García, J. P., Alzate, J. F. & Cabarcas, F. CLAME: a new alignment-based binning algorithm allows the genomic description of a novel *Xanthomonadaceae* from the Colombian Andes. *BMC Genomics* **19**, (2018).
35. Nurk, S., Meleshko, D., Korobeynikov, A. & Pevzner, P. A. MetaSPAdes: A new versatile metagenomic assembler. *Genome Res.* **27**, 824–834 (2017).
36. Zerbino, D. R. & Birney, E. Velvet: Algorithms for de novo short read assembly using de Bruijn graphs. *Genome Res.* **18**, 821–829 (2008).
37. Morgulis, A. *et al.* Database indexing for production MegaBLAST searches. *Bioinformatics* **24**, 1757–1764 (2008).
38. Menzel, P., Ng, K. L. & Krogh, A. Fast and sensitive taxonomic classification for metagenomics with Kaiju. *Nat. Commun.* **7**, 11257 (2016).
39. Hyatt, D. *et al.* Prodigal: Prokaryotic gene recognition and translation initiation site identification. *BMC Bioinformatics* **11**, 1–11 (2010).
40. Besemer, J. & Borodovsky, M. GeneMark: web software for gene finding in prokaryotes, eukaryotes and viruses. *Nucleic Acids Res.* **33**, W451–W454 (2005).
41. Ondov, B. D., Bergman, N. H. & Phillippy, A. M. Interactive metagenomic visualization in a Web browser. *BMC Bioinformatics* **12**, 1–10 (2011).
42. Bai, D. *et al.* EasyMetagenome: A user-friendly and flexible pipeline for shotgun metagenomic analysis in microbiome research. *iMeta* **4**, e70001 (2025).
43. Olm, M. R., Brown, C. T., Brooks, B. & Banfield, J. F. dRep: a tool for fast and accurate genomic comparisons that enables improved genome recovery from metagenomes through de-replication. *ISME J.* **11**, 2864–2868 (2017).
44. Kanehisa, M., Sato, Y. & Morishima, K. BlastKOALA and GhostKOALA: KEGG Tools for Functional Characterization of Genome and Metagenome Sequences. *J. Mol. Biol.* **428**, 726–731 (2016).
45. Huerta-Cepas, J. *et al.* eggNOG 5.0: a hierarchical, functionally and phylogenetically annotated orthology resource based on 5090 organisms and 2502 viruses. *Nucleic Acids Res.* **47**, D309–D314 (2019).
46. Zheng, J. *et al.* dbCAN3: automated carbohydrate-active enzyme and substrate annotation. *Nucleic Acids Res.* **51**, W115–W121 (2023).
47. Blanco-Míguez, A. *et al.* Extending and improving metagenomic taxonomic profiling with uncharacterized species using MetaPhlAn 4. *Nat. Biotechnol.* 1–12 (2023) doi:10.1038/s41587-023-01688-w.
48. Beghini, F. *et al.* Integrating taxonomic, functional, and strain-level profiling of diverse microbial communities with bioBakery 3. *eLife* **10**, e65088 (2021).
49. Liu, B., Zheng, D., Zhou, S., Chen, L. & Yang, J. VFDB 2022: a general classification scheme for bacterial virulence factors. *Nucleic Acids Res.* **50**, D912–D917 (2022).
50. Alcock, B. P. *et al.* CARD 2023: expanded curation, support for machine learning, and resistome prediction at the Comprehensive Antibiotic Resistance Database. *Nucleic Acids Res.* **51**, D690–D699 (2023).
51. Cavalcante, J. V. F., Dantas de Souza, I., Morais, D. A. A. & Dalmolin, R. J. S. EURYALE: A versatile Nextflow pipeline for taxonomic classification and functional annotation of metagenomics data. in *2024 IEEE Conference on Computational Intelligence in Bioinformatics and Computational Biology (CIBCB)* 1–7 (2024).
52. Morais, D. A. A., Cavalcante, J. V. F., Monteiro, S. S., Pasquali, M. A. B. & Dalmolin, R. J. S. MEDUSA: A Pipeline for Sensitive Taxonomic Classification and Flexible Functional Annotation of Metagenomic Shotgun Sequences. *Front. Genet.* **13**, (2022).
53. Chen, S., Zhou, Y., Chen, Y. & Gu, J. fastp: an ultra-fast all-in-one FASTQ preprocessor. *Bioinformatics* **34**, i884–i890 (2018).
54. Ewels, P., Magnusson, M., Lundin, S. & Käller, M. MultiQC: summarize analysis results for multiple tools and samples in a single report. *Bioinformatics* **32**, 3047–3048 (2016).
55. Buchfink, B., Reuter, K. & Drost, H.-G. Sensitive protein alignments at tree-of-life scale using

DIAMOND. *Nat. Methods* **18**, 366–368 (2021).
56. McCulloch, J. A. *et al.* JAMS - A framework for the taxonomic and functional exploration of microbiological genomic data. Preprint at https://doi.org/10.1101/2023.03.03.531026 (2023).
57. Bankevich, A. *et al.* SPAdes: A New Genome Assembly Algorithm and Its Applications to Single-Cell Sequencing. *J. Comput. Biol.* **19**, 455–477 (2012).
58. Seemann, T. Prokka: rapid prokaryotic genome annotation. *Bioinformatics* **30**, 2068–2069 (2014).
59. Zdobnov, E. M. & Apweiler, R. InterProScan – an integration platform for the signature-recognition methods in InterPro. *Bioinformatics* **17**, 847–848 (2001).
60. Churcheward, B., Millet, M., Bihouée, A., Fertin, G. & Chaffron, S. MAGNETO: An Automated Workflow for Genome-Resolved Metagenomics. *mSystems* **7**, (2022).
61. Köster, J. *et al.* Sustainable data analysis with Snakemake. *F1000Research* **10**, 33 (2021).
62. Wingett, S. W. & Andrews, S. FastQ Screen: A tool for multi-genome mapping and quality control. F1000Research 7, 1338, (2018).
63. Benoit, G. *et al.* Multiple comparative metagenomics using multiset k-mer counting. *PeerJ Comput. Sci.* **2**, e94 (2016).
64. Parks, D. H., Imelfort, M., Skennerton, C. T., Hugenholtz, P. & Tyson, G. W. CheckM: assessing the quality of microbial genomes recovered from isolates, single cells, and metagenomes. *Genome Res.* **25**, 1043–1055 (2015).
65. Steinegger, M. & Söding, J. Clustering huge protein sequence sets in linear time. *Nat. Commun.* **9**, 2542 (2018).
66. Li, W. & Godzik, A. Cd-hit: a fast program for clustering and comparing large sets of protein or nucleotide sequences. *Bioinformatics* **22**, 1658–1659 (2006).
67. Cantalapiedra, C. P., Hernández-Plaza, A., Letunic, I., Bork, P. & Huerta-Cepas, J. eggNOG-mapper v2: Functional Annotation, Orthology Assignments, and Domain Prediction at the Metagenomic Scale. *Mol. Biol. Evol.* **38**, 5825–5829 (2021).
68. Ruscheweyh, H.-J. *et al.* Cultivation-independent genomes greatly expand taxonomic-profiling capabilities of mOTUs across various environments. *Microbiome* **10**, 212 (2022).
69. Murovec, B., Deutsch, L. & Stres, B. Computational Framework for High-Quality Production and Large-Scale Evolutionary Analysis of Metagenome Assembled Genomes. *Mol. Biol. Evol.* **37**, 593–598 (2020).
70. Peng, Y., Leung, H. C. M., Yiu, S. M. & Chin, F. Y. L. IDBA-UD: a de novo assembler for single-cell and metagenomic sequencing data with highly uneven depth. *Bioinformatics* **28**, 1420–1428 (2012).
71. Kang, D. D., Froula, J., Egan, R. & Wang, Z. MetaBAT, an efficient tool for accurately reconstructing single genomes from complex microbial communities. *PeerJ* **3**, e1165 (2015).
72. Chaumeil, P. A., Mussig, A. J., Hugenholtz, P. & Parks, D. H. GTDB-Tk: a toolkit to classify genomes with the Genome Taxonomy Database. *Bioinformatics* **36**, 1925–1927 (2020).
73. Wu, Y.-W. ezTree: an automated pipeline for identifying phylogenetic marker genes and inferring evolutionary relationships among uncultivated prokaryotic draft genomes. *BMC Genomics* **19**, 921 (2018).
74. Jain, C., Rodriguez-R, L. M., Phillippy, A. M., Konstantinidis, K. T. & Aluru, S. High throughput ANI analysis of 90K prokaryotic genomes reveals clear species boundaries. *Nat. Commun.* **9**, 5114 (2018).
75. Zorrilla, F., Buric, F., Patil, K. R. & Zelezniak, A. metaGEM: reconstruction of genome scale metabolic models directly from metagenomes. *Nucleic Acids Res.* **49**, e126 (2021).
76. Li, H. & Durbin, R. Fast and accurate short read alignment with Burrows–Wheeler transform. *Bioinformatics* **25**, 1754–1760 (2009).
77. Machado, D., Andrejev, S., Tramontano, M. & Patil, K. R. Fast automated reconstruction of genome-scale metabolic models for microbial species and communities. *Nucleic Acids Res.* **46**, 7542–7553 (2018).
78. Zelezniak, A. *et al.* Metabolic dependencies drive species co-occurrence in diverse microbial communities. *Proc. Natl. Acad. Sci.* **112**, 6449–6454 (2015).
79. Lieven, C. *et al.* MEMOTE for standardized genome-scale metabolic model testing. *Nat. Biotechnol.* **38**, 272–276 (2020).
80. Page, A. J. *et al.* Roary: rapid large-scale prokaryote pan genome analysis. *Bioinformatics* **31**, 3691–3693 (2015).
81. Emiola, A. & Oh, J. High throughput in situ metagenomic measurement of bacterial replication at ultra-low sequencing coverage. *Nat. Commun.* **9**, 4956 (2018).
82. West, P. T., Probst, A. J., Grigoriev, I. V., Thomas, B. C. & Banfield, J. F. Genome-reconstruction for eukaryotes from complex natural microbial communities. *Genome Res.* **28**, 569–580 (2018).
83. Saary, P., Mitchell, A. L. & Finn, R. D. Estimating the quality of eukaryotic genomes recovered from metagenomic analysis with EukCC. *Genome Biol.* **21**, 244 (2020).
84. Milanese, A. *et al.* Microbial abundance, activity and population genomic profiling with mOTUs2. *Nat. Commun.* **10**, 1014 (2019).
85. Shaban, B. et al. MetaGenePipe: An Automated, Portable Pipeline for Contig-based Functional and Taxonomic Analysis. The Journal of Open Source Software 8, 4851 (2023).
86. Aramaki, T. *et al.* KofamKOALA: KEGG Ortholog assignment based on profile HMM and adaptive score threshold. *Bioinformatics* **36**, 2251–2252 (2020).
87. Kieser, S., Brown, J., Zdobnov, E. M., Trajkovski, M. & McCue, L. A. ATLAS: A Snakemake workflow for assembly, annotation, and genomic binning of metagenome sequence data. *BMC Bioinformatics* **21**, 1–8 (2020).
88. Líndez, P. P. *et al.* Adversarial and variational autoencoders improve metagenomic binning. *Commun. Biol.* **6**, 1–10 (2023).
89. Manni, M., Berkeley, M. R., Seppey, M. & Zdobnov, E. M. BUSCO: Assessing Genomic Data Quality and Beyond. *Curr. Protoc.* **1**, e323 (2021).
90. Orakov, A. *et al.* GUNC: detection of chimerism and contamination in prokaryotic genomes. *Genome Biol.* **22**, 1–19 (2021).
91. Shaffer, M. *et al.* DRAM for distilling microbial metabolism to automate the curation of microbiome function. *Nucleic Acids Res.* **48**, 8883–8900 (2020).
92. Salazar, V. W. *et al.* Metaphor—A workflow for streamlined assembly and binning of metagenomes. *GigaScience* **12**, 1–12 (2023).
93. Mikheenko, A., Saveliev, V. & Gurevich, A. MetaQUAST: evaluation of metagenome assemblies. *Bioinformatics* **32**, 1088–1090 (2016).
94. Li, H. Minimap2: pairwise alignment for nucleotide sequences. *Bioinformatics* **34**, 3094–3100 (2018).

95. Dröge, J., Gregor, I. & McHardy, A. C. Taxator-tk: precise taxonomic assignment of metagenomes by fast approximation of evolutionary neighborhoods. *Bioinformatics* **31**, 817–824 (2015).
96. Krapohl, J. & Pickett, B. E. SnakeWRAP: a Snakemake workflow to facilitate automated processing of metagenomic data through the metaWRAP pipeline. *F1000Research* **11**, (2022).
97. Ziemski, M. *et al.* MOSHPIT: accessible, reproducible metagenome data science on the QIIME 2 framework. Preprint at https://doi.org/10.1101/2025.01.27.635007 (2025).
98. Bolyen, E. *et al.* Reproducible, interactive, scalable and extensible microbiome data science using QIIME 2. *Nat. Biotechnol.* **37**, 852–857 (2019).
99. Narayanasamy, S. *et al.* IMP: a pipeline for reproducible reference-independent integrated metagenomic and metatranscriptomic analyses. *Genome Biol.* **17**, 260 (2016).
100. Kopylova, E., Noé, L. & Touzet, H. SortMeRNA: fast and accurate filtering of ribosomal RNAs in metatranscriptomic data. *Bioinformatics* **28**, 3211–3217 (2012).
101. Bushnell, B. BBMap: A Fast, Accurate, Splice-Aware Aligner. LBL Publications, (2014).
102. Sunagawa, S. *et al.* Metagenomic species profiling using universal phylogenetic marker genes. *Nat. Methods* **10**, 1196–1199 (2013).
103. Schudoma, C. Source code for: gff_quantifier. https://github.com/cschu/gff_quantifier (2023).
104. Tadrent, N. *et al.* SnakeMAGs: a simple, efficient, flexible and scalable workflow to reconstruct prokaryotic genomes from metagenomes. *F1000Research* **11**, 1522 (2023).
105. Eren, A. M., Vineis, J. H., Morrison, H. G. & Sogin, M. L. A Filtering Method to Generate High Quality Short Reads Using Illumina Paired-End Technology. *PLOS ONE* **8**, e66643 (2013).
106. Aroney, S. T. N. *et al.* CoverM: read alignment statistics for metagenomics. *Bioinformatics* **41**, btaf147 (2025).
107. Schmidt, T. S. B. *et al.* SPIRE: a Searchable, Planetary-scale mIcrobiome REsource. *Nucleic Acids Res.* **52**, D777–D783 (2024).
108. Coelho, L. P. *et al.* NG-meta-profiler: fast processing of metagenomes using NGLess, a domain-specific language. *Microbiome* **7**, 84 (2019).
109. Seemann, T. Source code for: Barrnap-Bacterial ribosomal RNA predictor. https://github.com/tseemann/shovill (2018).
110. Seemann, T. Source code for: ABRicate-Mass screening of contigs for antimicrobial and virulence genes. https://github.com/tseemann/abricate (2020).
111. Santos-Júnior, C. D., Pan, S., Zhao, X.-M. & Coelho, L. P. Macrel: antimicrobial peptide screening in genomes and metagenomes. *PeerJ* **8**, e10555 (2020).
112. Göbel, D., Stoye, J., Sczyrba, A., & Beckstette, M. The Cloud-based Workflow Manager (CloWM) - An integrated platform for highly scalable workflow execution. German Conference on Bioinformatics 2024 (GCB), Bielefeld. Zenodo. https://doi.org/10.5281/zenodo.14039069 (2024).
113. Clarke, E. L. *et al.* Sunbeam: An extensible pipeline for analyzing metagenomic sequencing experiments. *Microbiome* **7**, 1–13 (2019).
114. Wood, D. E. & Salzberg, S. L. Kraken: ultrafast metagenomic sequence classification using exact alignments. *Genome Biol.* **15**, R46 (2014).
115. Peng, K. *et al.* Benchmarking of analysis tools and pipeline development for nanopore long-read metagenomics. *Sci. Bull.* **70**, 1591–1595 (2025).
116. Kolmogorov, M. *et al.* metaFlye: scalable long-read metagenome assembly using repeat graphs. *Nat. Methods* **17**, 1103–1110 (2020).
117. Bertrand, D. *et al.* Hybrid metagenomic assembly enables high-resolution analysis of resistance determinants and mobile elements in human microbiomes. *Nat. Biotechnol.* **37**, 937–944 (2019).
118. Kajitani, R. *et al.* MetaPlatanus: a metagenome assembler that combines long-range sequence links and species-specific features. *Nucleic Acids Res.* **49**, e130 (2021).
119. Hu, J., Fan, J., Sun, Z. & Liu, S. NextPolish: a fast and efficient genome polishing tool for long-read assembly. *Bioinformatics* **36**, 2253–2255 (2020).
120. Asnicar, F. *et al.* Precise phylogenetic analysis of microbial isolates and genomes from metagenomes using PhyloPhlAn 3.0. *Nat. Commun.* **11**, 1–10 (2020).
121. Kim, D., Song, L., Breitwieser, F. P. & Salzberg, S. L. Centrifuge: rapid and sensitive classification of metagenomic sequences. *Genome Res.* **26**, 1721–1729 (2016).
122. Portik, D. M. *et al.* Highly accurate metagenome-assembled genomes from human gut microbiota using long-read assembly, binning, and consolidation methods. Preprint at https://doi.org/10.1101/2024.05.10.593587 (2024).
123. Cheng, H., Concepcion, G. T., Feng, X., Zhang, H. & Li, H. Haplotype-resolved de novo assembly using phased assembly graphs with hifiasm. *Nat. Methods* **18**, 170–175 (2021).
124. Maurice, N., Lemaitre, C., Vicedomini, R. & Frioux, C. Mapler: a pipeline for assessing assembly quality in taxonomically rich metagenomes sequenced with HiFi reads. *Bioinformatics* **41**, btaf334 (2025).
125. Benoit, G. *et al.* High-quality metagenome assembly from long accurate reads with metaMDBG. *Nat. Biotechnol.* **42**, 1378–1383 (2024).
126. Mapleson, D., Garcia Accinelli, G., Kettleborough, G., Wright, J. & Clavijo, B. J. KAT: a K-mer analysis toolkit to quality control NGS datasets and genome assemblies. *Bioinformatics* **33**, 574–576 (2017).
127. Liu, L., Yang, Y., Deng, Y. & Zhang, T. Nanopore long-read-only metagenomics enables complete and high-quality genome reconstruction from mock and complex metagenomes. *Microbiome* **10**, 209 (2022).
128. Vaser, R., Sovic, I., Nagarajan, N. & Sikic, M. Fast and accurate de novo genome assembly from long uncorrected reads. Genome Res. 27, 737-746 (2017).
129. Walker, B. J. *et al.* Pilon: An Integrated Tool for Comprehensive Microbial Variant Detection and Genome Assembly Improvement. *PLOS ONE* **9**, e112963 (2014).
130. Stewart, R. D. *et al.* Compendium of 4,941 rumen metagenome-assembled genomes for rumen microbiome biology and enzyme discovery. *Nat. Biotechnol.* **37**, 953–961 (2019).
131. The UniProt Consortium. UniProt: the Universal Protein Knowledgebase in 2025. *Nucleic Acids Res.* **53**, D609–D617 (2025).
132. Kieft, K., Zhou, Z. & Anantharaman, K. VIBRANT: automated recovery, annotation and curation of microbial viruses, and evaluation of viral community function from genomic sequences. *Microbiome* **8**, 90 (2020).
133. Kieft, K. & Anantharaman, K. Deciphering Active Prophages from Metagenomes. *mSystems* **7**, e00084-22 (2022).
134. Cornet, L. *et al.* The GEN-ERA toolbox: unified and reproducible workflows for research in microbial genomics. *GigaScience* **12**, 1–10 (2022).
135. Brůna, T., Hoff, K. J., Lomsadze, A., Stanke, M. & Borodovsky, M. BRAKER2: automatic eukaryotic genome annotation with GeneMark-EP+ and

AUGUSTUS supported by a protein database. *NAR Genomics Bioinforma.* **3**, lqaa108 (2021).
136. Meunier, L., Baurain, D. & Cornet, L. AMAW: automated gene annotation for non-model eukaryotic genomes. F1000Research 12, 186 (2023).
137. Belmann, P. *et al.* Metagenomics-Toolkit: the flexible and efficient cloud-based metagenomics workflow featuring machine learning-enabled resource allocation. *NAR Genomics Bioinforma.* **7**, lqaf093 (2025).
138. Mainguy, J. *et al.* metagWGS, a comprehensive workflow to analyze metagenomic data using Illumina or PacBio HiFi reads. Preprint at https://doi.org/10.1101/2024.09.13.612854 (2024).
139. Mainguy, J. & Hoede, C. Binette: a fast and accurate bin refinement tool to construct high quality Metagenome Assembled Genomes. *J. Open Source Softw.* **9**, 6782 (2024).
140. Hildebrand, F. *et al.* Dispersal strategies shape persistence and evolution of human gut bacteria. *Cell Host Microbe* **29**, 1167-1176.e9 (2021).
141. Kolmogorov, M., Yuan, J., Lin, Y. & Pevzner, P. A. Assembly of long, error-prone reads using repeat graphs. *Nat. Biotechnol.* **37**, 540–546 (2019).
142. Liu, C.-C. *et al.* MetaDecoder: a novel method for clustering metagenomic contigs. *Microbiome* **10**, 46 (2022).
143. Parks, D. H. *et al.* GTDB: an ongoing census of bacterial and archaeal diversity through a phylogenetically consistent, rank normalized and complete genome-based taxonomy. *Nucleic Acids Res.* **50**, D785–D794 (2022).
144. Kanehisa, M., Sato, Y., Kawashima, M., Furumichi, M. & Tanabe, M. KEGG as a reference resource for gene and protein annotation. *Nucleic Acids Res.* **44**, D457–D462 (2016).
145. Overbeek, R. *et al.* The SEED and the Rapid Annotation of microbial genomes using Subsystems Technology (RAST). *Nucleic Acids Res.* **42**, D206 (2014).
146. Drula, E. *et al.* The carbohydrate-active enzyme database: functions and literature. *Nucleic Acids Res.* **50**, D571–D577 (2022).
147. Saier, M. H., Jr *et al.* The Transporter Classification Database (TCDB): 2021 update. *Nucleic Acids Res.* **49**, D461–D467 (2021).
148. Cokelaer, T., Desvillechabrol, D., Legendre, R. & Cardon, M. 'Sequana': a Set of Snakemake NGS pipelines. *J. Open Source Softw.* **2**, 352 (2017).
149. Espinoza, J. L. *et al.* Unveiling the microbial realm with VEBA 2.0: a modular bioinformatics suite for end-to-end genome-resolved prokaryotic, (micro)eukaryotic and viral multi-omics from either short- or long-read sequencing. *Nucleic Acids Res.* **52**, e63 (2024).
150. Suzek, B. E. *et al.* UniRef clusters: a comprehensive and scalable alternative for improving sequence similarity searches. *Bioinformatics* **31**, 926–932 (2015).
151. Zdouc, M. M. *et al.* MIBiG 4.0: advancing biosynthetic gene cluster curation through global collaboration. *Nucleic Acids Res.* **53**, D678–D690 (2025).
152. Mistry, J. *et al.* Pfam: The protein families database in 2021. *Nucleic Acids Res.* **49**, D412–D419 (2021).
153. Feldgarden, M. *et al.* AMRFinderPlus and the Reference Gene Catalog facilitate examination of the genomic links among antimicrobial resistance, stress response, and virulence. *Sci. Rep.* **11**, 12728 (2021).
154. Eberhardt, R. Y. *et al.* AntiFam: a tool to help identify spurious ORFs in protein annotation. *Database* **2012**, bas003 (2012).
155. Newell, R. J. P., Aroney, S. T. N., Zaugg, J., Sternes, P., Tyson, G. W., & Woodcroft, B. J. Aviary: Hybrid assembly and genome recovery from metagenomes with Aviary (v0.12.0). Zenodo. https://doi.org/10.5281/zenodo.15208119 (2025).
156. Wick, R. R., Judd, L. M., Gorrie, C. L. & Holt, K. E. Unicycler: Resolving bacterial genome assemblies from short and long sequencing reads. *PLOS Comput. Biol.* **13**, e1005595 (2017).
157. Newell, R. J. P., Tyson, G. W., & Woodcroft, B. J. . Rosella: Metagenomic binning using UMAP and HDBSCAN (v0.5.3). Zenodo. https://doi.org/10.5281/zenodo.10460259 (2024).
158. Newell, R. J. P., McMaster, E. S., Craig, P., Boden, M., Tyson, G. W., & Woodcroft, B. J. Lorikeet: strain-resolved metagenome analysis using local reassembly (v0.8.2). Zenodo. https://doi.org/10.5281/zenodo.10275469 (2023).
159. Damme, R. van *et al.* Metagenomics workflow for hybrid assembly, differential coverage binning, metatranscriptomics and pathway analysis (MUFFIN). *PLOS Comput. Biol.* **17**, 1–13 (2021).
160. Grabherr, M. G. *et al.* Full-length transcriptome assembly from RNA-Seq data without a reference genome. *Nat. Biotechnol.* **29**, 644–652 (2011).
161. Patro, R., Duggal, G., Love, M. I., Irizarry, R. A. & Kingsford, C. Salmon provides fast and bias-aware quantification of transcript expression. *Nat. Methods* **14**, 417–419 (2017).
162. Krakau, S., Straub, D., Gourlé, H., Gabernet, G. & Nahnsen, S. nf-core/mag: a best-practice pipeline for metagenome hybrid assembly and binning. *NAR Genomics Bioinforma.* **4**, (2022).
163. Wick, R. R., Judd, L. M., Gorrie, C. L. & Holt, K. E. Completing bacterial genome assemblies with multiplex MinION sequencing. *Microb. Genomics* **3**, e000132 (2017).
164. Haveman, N. J. *et al.* Evaluating the lettuce metatranscriptome with MinION sequencing for future spaceflight food production applications. *Npj Micrograviy* **7**, 22 (2021).
165. De Coster, W. & Rademakers, R. NanoPack2: population-scale evaluation of long-read sequencing data. *Bioinformatics* **39**, btad311 (2023).
166. Schubert, M., Lindgreen, S. & Orlando, L. AdapterRemoval v2: rapid adapter trimming, identification, and read merging. *BMC Res. Notes* **9**, 88 (2016).
167. Antipov, D., Korobeynikov, A., McLean, J. S. & Pevzner, P. A. hybridSPAdes: an algorithm for hybrid assembly of short and long reads. *Bioinformatics* **32**, 1009–1015 (2016).
168. von Meijenfeldt, F. A. B., Arkhipova, K., Cambuy, D. D., Coutinho, F. H. & Dutilh, B. E. Robust taxonomic classification of uncharted microbial sequences and bins with CAT and BAT. *Genome Biol.* **20**, 217 (2019).
169. Levy Karin, E., Mirdita, M. & Söding, J. MetaEuk—sensitive, high-throughput gene discovery, and annotation for large-scale eukaryotic metagenomics. *Microbiome* **8**, 48 (2020).
170. Borry, M., Hübner, A., Rohrlach, A. B. & Warinner, C. PyDamage: automated ancient damage identification and estimation for contigs in ancient DNA de novo assembly. *PeerJ* **9**, e11845 (2021).
171. Karlicki, M., Antonowicz, S. & Karnkowska, A. Tiara: deep learning-based classification system for eukaryotic sequences. *Bioinformatics* **38**, 344–350 (2022).
172. Camargo, A. P. *et al.* Identification of mobile genetic elements with geNomad. *Nat. Biotechnol.* **42**, 1303–1312 (2024).
173. Almeida, F. M. de, Campos, T. A. de & Pappas, G. J. Scalable and versatile container-based pipelines for

de novo genome assembly and bacterial annotation. *F1000Research* **12**, 1205 (2023).
174. Koren, S. *et al.* Canu: scalable and accurate long-read assembly via adaptive k-mer weighting and repeat separation. *Genome Res.* **27**, 722–736 (2017).
175. Schwengers, O. *et al.* Bakta: Rapid and standardized annotation of bacterial genomes via alignment-free sequence identification. *Microb. Genomics* **7**, 000685 (2021).
176. Jolley, K. A. & Maiden, M. C. BIGSdb: Scalable analysis of bacterial genome variation at the population level. *BMC Bioinformatics* **11**, 595 (2010).
177. Graham, E. D., Heidelberg, J. F. & Tully, B. J. Potential for primary productivity in a globally-distributed bacterial phototroph. *ISME J.* **12**, 1861–1866 (2018).
178. Blin, K. *et al.* antiSMASH 7.0: new and improved predictions for detection, regulation, chemical structures and visualisation. *Nucleic Acids Res.* **51**, W46–W50 (2023).
179. Hu, K., Huang, N., Zou, Y., Liao, X. & Wang, J. MultiNanopolish: refined grouping method for reducing redundant calculations in Nanopolish. *Bioinformatics* **37**, 2757–2760 (2021).
180. Tamames, J. & Puente-Sánchez, F. SqueezeMeta, a highly portable, fully automatic metagenomic analysis pipeline. *Front. Microbiol.* **10**, 3349 (2019).
181. Bushmanova, E., Antipov, D., Lapidus, A. & Prjibelski, A. D. rnaSPAdes: a de novo transcriptome assembler and its application to RNA-Seq data. *GigaScience* **8**, giz100 (2019).
182. Caspi, R. *et al.* The MetaCyc database of metabolic pathways and enzymes - a 2019 update. *Nucleic Acids Res.* **48**, D445–D453 (2020).
183. Olson, R. D. *et al.* Introducing the Bacterial and Viral Bioinformatics Resource Center (BV-BRC): a resource combining PATRIC, IRD and ViPR. *Nucleic Acids Res.* **51**, D678–D689 (2023).
184. Gillespie, J. J. *et al.* PATRIC: the Comprehensive Bacterial Bioinformatics Resource with a Focus on Human Pathogenic Species. *Infect. Immun.* **79**, 4286–4298 (2011).
185. Brettin, T. *et al.* RASTtk: A modular and extensible implementation of the RAST algorithm for building custom annotation pipelines and annotating batches of genomes. *Sci. Rep.* **5**, 8365 (2015).
186. Wang, S., Sundaram, J. P. & Spiro, D. VIGOR, an annotation program for small viral genomes. *BMC Bioinformatics* **11**, 451 (2010).
187. The Galaxy Community *et al.* The Galaxy platform for accessible, reproducible and collaborative biomedical analyses: 2022 update. *Nucleic Acids Res.* **50**, W345–W351 (2022).
188. Kalantar, K. L. *et al.* IDseq—An open source cloud-based pipeline and analysis service for metagenomic pathogen detection and monitoring. *GigaScience* **9**, giaa111 (2020).
189. Chen, I.-M. A. *et al.* The IMG/M data management and analysis system v.7: content updates and new features. *Nucleic Acids Res.* **51**, D723–D732 (2023).
190. Kanehisa, M., Furumichi, M., Sato, Y., Ishiguro-Watanabe, M. & Tanabe, M. KEGG: integrating viruses and cellular organisms. *Nucleic Acids Res.* **49**, D545–D551 (2021).
191. Galperin, M. Y. *et al.* COG database update 2024. *Nucleic Acids Res.* **53**, D356–D363 (2025).
192. Haft, D. H. *et al.* TIGRFAMs and Genome Properties in 2013. *Nucleic Acids Res.* **41**, D387–D395 (2013).
193. Arkin, A. P. *et al.* KBase: The United States Department of Energy Systems Biology Knowledgebase. *Nat. Biotechnol.* **36**, 566–569 (2018).
194. Seaver, S. M. D. *et al.* The ModelSEED Biochemistry Database for the integration of metabolic annotations and the reconstruction, comparison and analysis of metabolic models for plants, fungi and microbes. *Nucleic Acids Res.* **49**, D575–D588 (2021).
195. Richardson, L. *et al.* MGnify: the microbiome sequence data analysis resource in 2023. *Nucleic Acids Res.* **51**, D753–D759 (2023).
196. Finn, R. D., Clements, J. & Eddy, S. R. HMMER web server: interactive sequence similarity searching. *Nucleic Acids Res.* **39**, W29–W37 (2011).
197. Weber, N. *et al.* Nephele: a cloud platform for simplified, standardized and reproducible microbiome data analysis. *Bioinformatics* **34**, 1411–1413 (2018).
198. Standeven, F. J., Dahlquist-Axe, G., Speller, C. F., Meehan, C. J. & Tedder, A. An efficient pipeline for creating metagenomic-assembled genomes from ancient oral microbiomes. Preprint at https://doi.org/10.1101/2024.09.18.613623 (2024).
199. Jónsson, H., Ginolhac, A., Schubert, M., Johnson, P. L. F. & Orlando, L. mapDamage2.0: fast approximate Bayesian estimates of ancient DNA damage parameters. *Bioinformatics* **29**, 1682–1684 (2013).
200. Zhao, D. *et al.* Eukfinder: a pipeline to retrieve microbial eukaryote genome sequences from metagenomic data. *mBio* **16**, e00699-25 (2025).
201. Van Nguyen, H. & Lavenier, D. PLAST: parallel local alignment search tool for database comparison. *BMC Bioinformatics* **10**, 329 (2009).
202. Lin, H.-H. & Liao, Y.-C. Accurate binning of metagenomic contigs via automated clustering sequences using information of genomic signatures and marker genes. *Sci. Rep.* **6**, 24175 (2016).
